# Sulfur-Ligated [2Fe-2C] Clusters as Synthetic Model Systems for Nitrogenase

**DOI:** 10.1021/acs.inorgchem.2c03693

**Published:** 2023-01-30

**Authors:** Sivathmeehan Yogendra, Daniel W. N. Wilson, Anselm W. Hahn, Thomas Weyhermüller, Casey Van Stappen, Patrick Holland, Serena DeBeer

**Affiliations:** † Max Planck Institute for Chemical Energy Conversion, Stiftstrasse 34-36, 45470 Mülheim an der Ruhr, Germany; ‡ Department of Chemistry, Yale University, 225 Prospect Street, New Haven, Connecticut 06520, United States

## Abstract

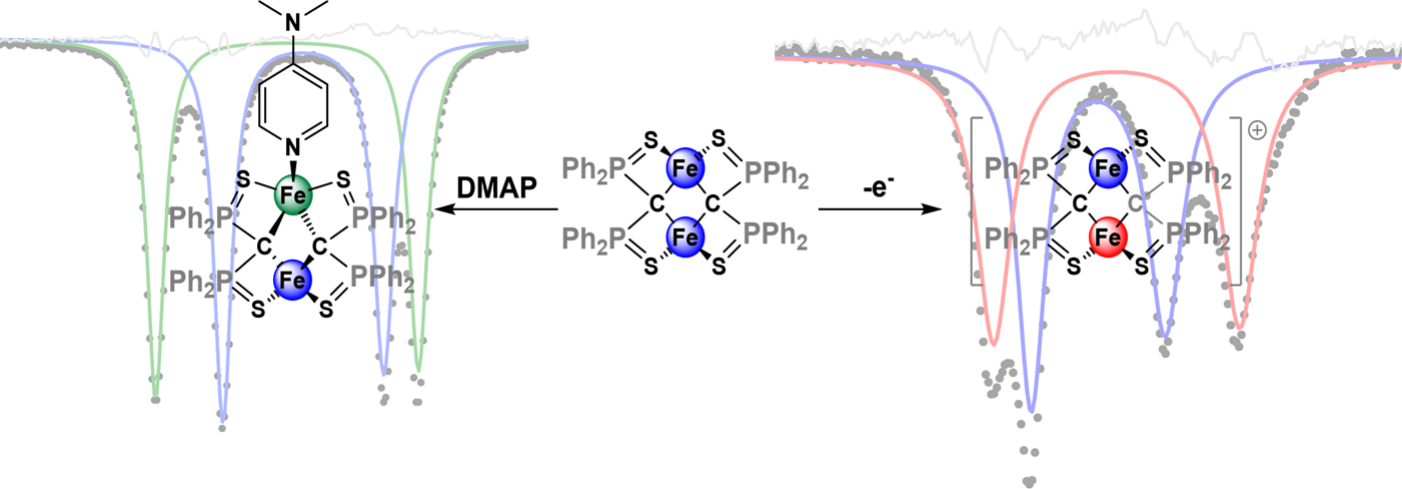

Metal clusters featuring carbon and sulfur donors have coordination environments comparable to the active site of nitrogenase enzymes. Here, we report a series of di-iron clusters supported by the dianionic yldiide ligands, in which the Fe sites are bridged by two μ^2^-C atoms and four pendant S donors.The [L_2_Fe_2_] (L = {[Ph_2_P(S)]_2_C}^2–^) cluster is isolable in two oxidation levels, all-ferrous Fe_2_^II^ and mixed-valence Fe^II^Fe^III^. The mixed-valence cluster displays two peaks in the Mössbauer spectra, indicating slow electron transfer between the two sites. The addition of the Lewis base 4-dimethylaminopyridine to the Fe_2_^II^ cluster results in coordination with only one of the two Fe sites, even in the presence of an excess base. Conversely, the cluster reacts with 8 equiv of isocyanide ^*t*^BuNC to give a monometallic complex featuring a new C–C bond between the ligand backbone and the isocyanide. The electronic structure descriptions of these complexes are further supported by X-ray absorption and resonant X-ray emission spectroscopies.

## Abstract 2

Here, we investigated [L_2_Fe_2_] (L = {[Ph_2_P(S)]_2_C}^2−^) from the perspective of the nitrogenase cofactor, FeMco. The cluster is isolable in two oxidation levels, the all-ferrous Fe_2_^II^ and the mixed-valence Fe^II^Fe^III^. The mixed-valence cluster displays slow electron transfer between the two sites. The addition of 4-dimethylaminopyridine to the Fe_2_^II^ cluster results in coordination with only one of the two Fe sites. Conversely, the cluster reacts with 8 equiv of isocyanide ^*t*^BuNC to give a monometallic complex featuring a new C−C bond between the ligand backbone and the isocyanide. The electronic structure descriptions of these complexes are further supported by X-ray absorption and resonant X-ray emission spectroscopies.

## Introduction

While nitrogen is an essential element, its most abundant form N_2_ is relatively inert, and therefore it must be converted to more reactive forms for industrial or biological use.^1^ In nature, nitrogenase enzymes reduce N_2_ to NH_3_ at atmospheric pressure and ambient temperature using the specialized iron–sulfur clusters known as FeMco (M = Mo, V, and Fe).^2−4^ All three of the nitrogenases contain a central μ^6^-carbide (C^4–^) bridging six-belt Fe atoms,^5−7^ one of which is the proposed binding site for N_2_ during nitrogen fixation.^8,9^ While the role of the carbide is currently unknown, the extensive biosynthetic pathway for its incorporation into the cofactor implies that it plays a crucial role.^10−12^ One proposed role of the carbide is to provide structural support that stabilizes the cluster during displacement of a belt sulfide, which serves to open a binding site for the substrate at a belt Fe. Evidence for this proposal includes crystallographic identification of species in which CO is bound to FeMco (M = V and Mo) in the space vacated by the loss of a bridging sulfide.^8,13,14^ In another model, the sulfides remain coordinated, but the carbide stabilizes a trigonal–bipyramidal N_2_ intermediate; supporting this idea, a series of N_2_ reduction catalysts feature an Fe site with an axial carbon ligand trans to the N_2_-binding site.^15^ Crystallographic studies on the nitrogenase enzymes have not yet shown a cofactor with a nitrogen-derived substrate bound,^16,17^ and kinetic/spectroscopic studies suggest that the initial N_2_ complex is trapped rapidly.^18^ A range of computational studies disagree on the preferred binding site for N_2_.^19−22^ Additionally, the carbide may play a role in electron transfer/delocalization between Fe sites, facilitating the migration of electrons in a way that sulfur or other biologically available elements cannot.^23−25^

**Figure 1:**
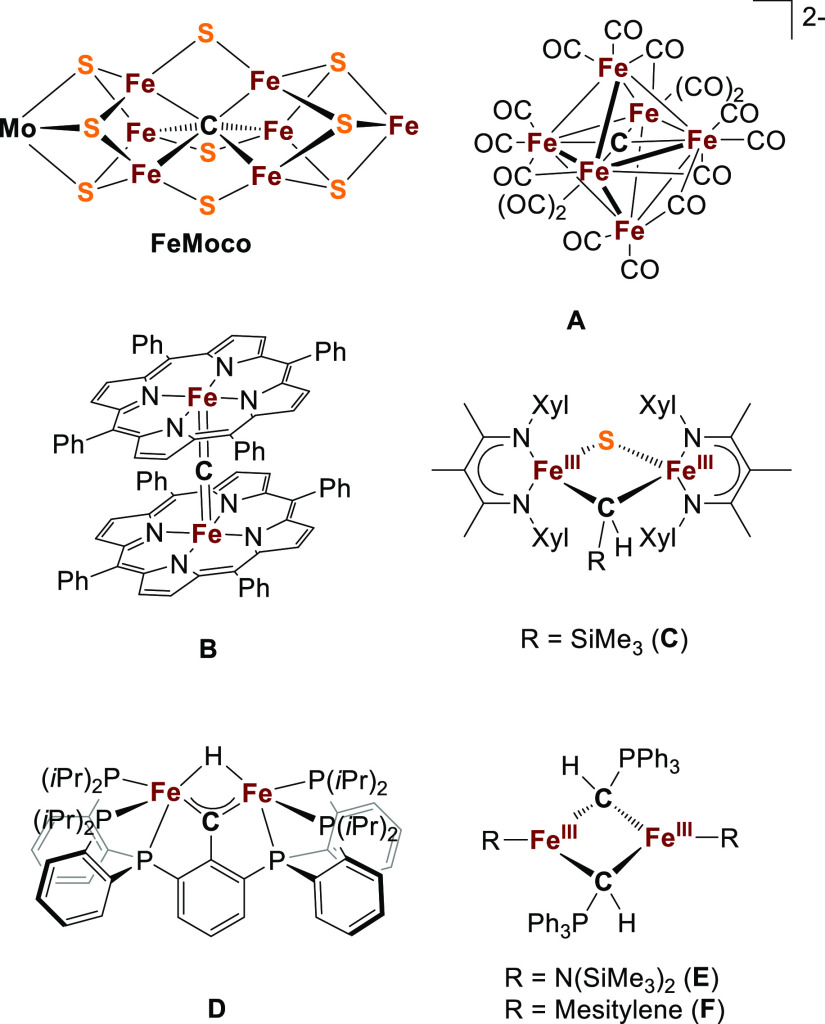
Selected examples of model complexes relevant to the study of FeMco clusters.

**Figure 2:**
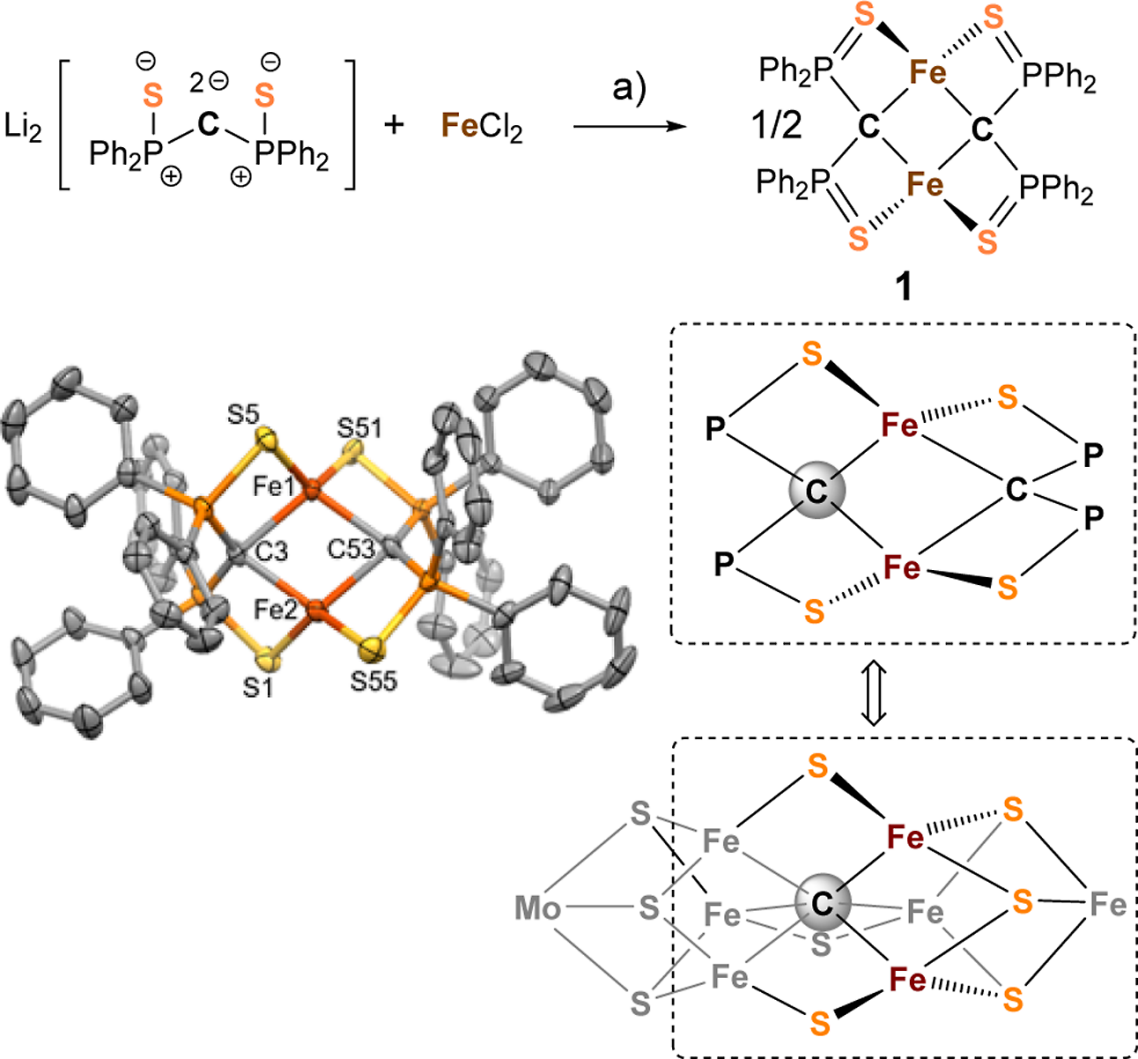
Top: Reaction of bis(diphenylthiophosphinoyl)methanediide with FeCl_2_ to give 1; (a) THF, −35 °C to rt, 30 min, 58%. Left bottom: molecular structure of 1 (hydrogen atoms are omitted for clarity and thermal ellipsoids are displayed at 50% probability): selected bond lengths in angstroms and angles in degrees: Fe1···Fe2 2.5519(4), Fe1–C3 2.100(2), Fe1–C53 2.102(2), Fe2–C3 2.101(2), Fe2–C53 2.092(2), Fe1–S5 2.3589(5), Fe1–S55, 2.3569(5), Fe2–S1 2.3798(6), Fe2–S51 2.3530(5), Fe1–C3–Fe2, 74.82(5), Fe1–C53–Fe2 74.85(6), C3–Fe1–C53 104.95(7), and C3–Fe2–C53 105.27(7). Right bottom: comparison of the structural topology of 1 with that of the belt iron atoms in the FeMoco.

One way of addressing these coordination chemistry questions is by preparing isolable complexes that share features with potential intermediates. These model complexes can serve as spectroscopic benchmarks for the cofactor as well as enable synthetic chemists to test reactivity modes in well-characterized simpler systems.^26,27^ However, it is difficult to accurately mimic the μ^6^-carbide in FeMco. Carbide ligands to Fe are known in iron carbonyl clusters, which can feature μ^3^-, μ^5^-, or μ^6^-C ligands, for example, **A** in Figure 1.^28−32^ However, the strong-field ligand environments result in low-spin electronic configurations, which give different bond lengths and spectroscopic features from the high-spin sites expected in the sulfur-rich environment of FeMco. Thus, these are of limited use as models of FeMco. The only other class of carbide-containing Fe complexes are diamagnetic and supported by porphyrin ligands (**B**).^33^ As an alternative, chemists have also prepared ligands that have other C donors as mimics for the carbide, and particular interest has been in ligands that also have S donors like nitrogenase.^26,34,35^ There have been recent examples of bimetallic complexes bridged by μ^2^-alkylidene and -alkylidyne ligands (**C**–**F**); however, these include nonbiological donor atoms in the coordination sphere of Fe, making it difficult to deconvolute the effect of the carbon from the other ligands.^36−39^ We sought a complex having a more nitrogenase-relevant S/C coordination environment. To this end, we selected the previously reported cluster **1**, which features two Fe sites bridged by an yldiide ligand (Figure 2).^40^

**1** is a rare example of a di-iron complex containing only μ^2^-C and sulfur ligands, which also has tetrahedral, high-spin Fe sites; these properties suggest that it can give useful insights into the environments expected for Fe in nitrogenase. In this manuscript, we explore the spectroscopy of **1**, as well as the products from one-electron oxidation and external ligand coordination.

## Results and Discussion

**Figure 3:**
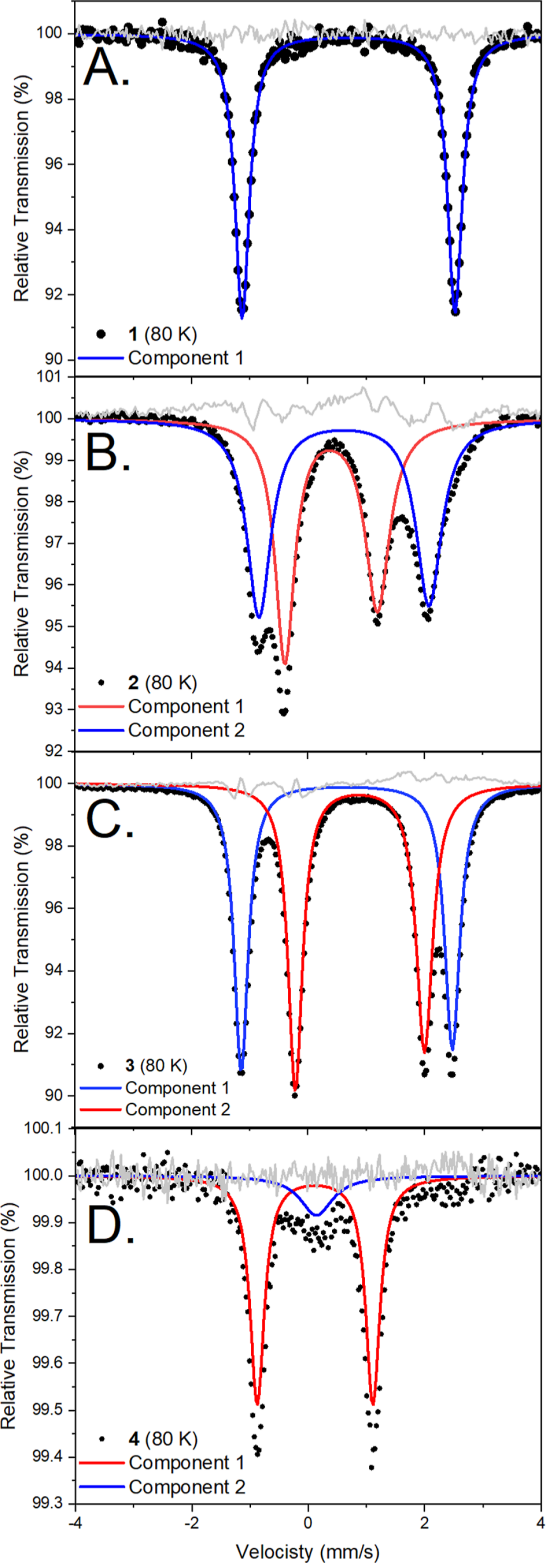
Zero-field ^57^Fe Mössbauer spectra of solid 1, 2, 3, and 4 recorded at 80 K. The solid lines represent the fit for 1 (A) with δ = 0.70 mm s^–1^ and Δ*E*_Q_ = 3.65 mm s^–1^; for 2 (B) with δ = 0.40 mm s^–1^ and Δ*E*_Q_ = 1.59 mm s^–1^ (red) (Fe^III^) and δ = 0.62 mm s^–1^ and Δ*E*_Q_ = 2.92 mm s^–1^ (blue) (Fe^II^) site; for 3, (C) with δ = 0.67 mm s^–1^ and Δ*E*_Q_ = 3.63 mm s^–1^ for the tetracoordinated Fe^II^ site (red) and δ = 0.89 mm s^–1^ and Δ*E*_Q_ = 2.22 mm s^–1^ for the pentacoordinated Fe^II^ site (blue); and for 4, (D) with δ = 0.12 mm s^–1^ and Δ*E*_Q_ = 1.98 mm s^–1^ (red). A second component (blue) represents 14% of the signal and is associated with a background signal in the spectrometer. Gray lines represent the residuals (model—data).

**1** was prepared via a simplified version of the reported literature procedure, a direct reaction of bis(diphenylthiophosphinoyl)methanediide with FeCl_2_ (Figure 2). The identity was verified by comparison to the reported NMR spectra, which feature paramagnetically shifted ^1^H NMR signals and a featureless ^31^P NMR spectrum, and also by single-crystal X-ray diffraction.^40^ Further characterization of **1** was not included in the original publication and will be discussed herein. The UV–vis spectrum of **1** reveals a distinct absorption band at 595 nm (Figure S11, see the Supporting Information). The ^57^Fe Mössbauer spectrum of **1** recorded at 80 K (Figure 3A) and displays a doublet with δ = 0.70 mm s^–1^ and Δ*E*_Q_ = 3.65 mm s^–1^, indicating that there are equivalent high-spin Fe^II^ sites. Slightly higher isomer shifts were reported for Fe^II^ β-diketiminato-substituted (δ = 0.77 mm s^–1^ and Δ*E*_Q_ = 2.24 mm s^–1^) and bis(benzimidazolato)-substituted (δ = 0.79 mm s^–1^ and Δ*E*_Q_ = 2.67 mm s^–1^) [2Fe–2S] clusters that are iron (II).^41−43^ Significantly lower isomer shifts were observed for the C-bridged dimers **E** and **F** (**E**: δ = 0.35 mm s^–1^ and Δ*E*_Q_ = 1.75 mm s^–1^; **F**: δ = 0.24 mm s^–1^ and Δ*E*_Q_ = 1.95 mm s^–1^), explained by the higher coordination number (CN) in **1** [CN(**1**) = 4 *vs* CN(**E**, **F**) = 3].^36^

The molecular structure of **1** determined by single-crystal X-ray diffraction agrees with the literature report.^40^ Its coordination environment is reminiscent of the belt iron atoms in the FeMoco, where a one-edge sulfide ligand is replaced by an yldiide ligand (Figure 2).^44^ Some structural similarity is evident by the comparable Fe···Fe distance in **1** of 2.5519(4) Å to the Fe···Fe distances observed for the belt Fe sites in the FeMoco (av 2.62 Å). However, the Fe–C [av 2.099(7) Å] and Fe–S [av 2.362(2) Å] bond lengths in **1** are longer than those in the FeMoco (Fe–C av 2.00 Å, Fe-μ^2^-S av 2.22 Å, and Fe-μ^3^-S av 2.26 Å), likely due to the lower charge localization on the “PS” moiety of L in comparison to the formally S^2–^ in the FeMoco.^45,46^ The acute Fe–C–Fe angles [av 74.9(1)°] are comparable to those in the FeMoco (Fe–C–Fe av 82°). Both the C–P bonds [1.72(1) Å] and the P–S bonds [2.027 (1) Å] are shorter than the sum of the covalent radii for single bonds (covalent radii for single bonds: C–P = 1.86 Å; P–S = 2.14 Å) but longer than double bonds (C–P = 1.69 Å; P–S = 1.96 Å),^47^ indicating partial delocalization of the lone pairs at the C and S atoms by negative hyperconjugation.^48,49^ The geometry at the Fe atoms in **1** is intermediate between tetrahedral and trigonal monopyramidal, giving calculated τ_4_-values of 0.33 and 0.26 (ideal tetrahedron: τ = 0 and ideal trigonal pyramid: τ = 1).^50^ This geometry is similar to that of the belt iron atoms of the FeMoco (av τ_4_ = 0.46), although not as pyramidalized.^51,52^

**Scheme 1:**
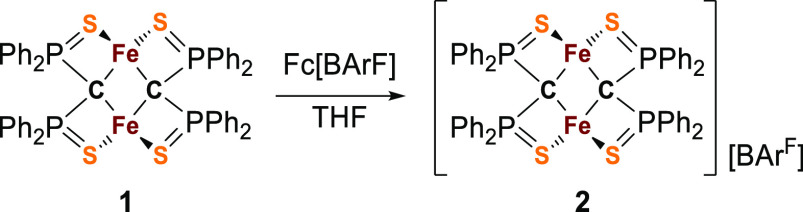
Oxidation of **1** to Give the Mixed-Valent Cluster **2**

The redox chemistry of **1** was investigated by cyclic voltammetry in CH_2_Cl_2_ at 255 K using [Bu_4_N]PF_6_ as a supporting electrolyte. Reversible oxidation is observed at *E*_1/2_ = −0.62 V corresponding to the formation of the mixed-valent cluster **2** (Figure S14). The complex is thus much more difficult to oxidize than clusters **A** (*E* = −2.55 V) and **B** (*E* = −2.10 V).^41−43^ We attribute this difference to the neutral charge in this di-iron(II) complex, whereas the literature systems are anionic in the di-iron(II) state. Cluster **2** can be synthesized in the pure form by treating diferrous **1** with [Cp_2_Fe][PF_6_] or [Cp_2_Fe][BArF] {[BArF]^−^ = [B(C_6_H_3_(CF_3_)_2_)_4_]^−^} to give the new monocation as a PF_6_^–^ or BArF^–^ salt (yield of **2**[BArF] = 77%). Both salts slowly decompose in solution and the solid state, but the BArF^–^ salt is somewhat more stable than the PF_6_ salt (Schemes 1).

**Scheme 2:**
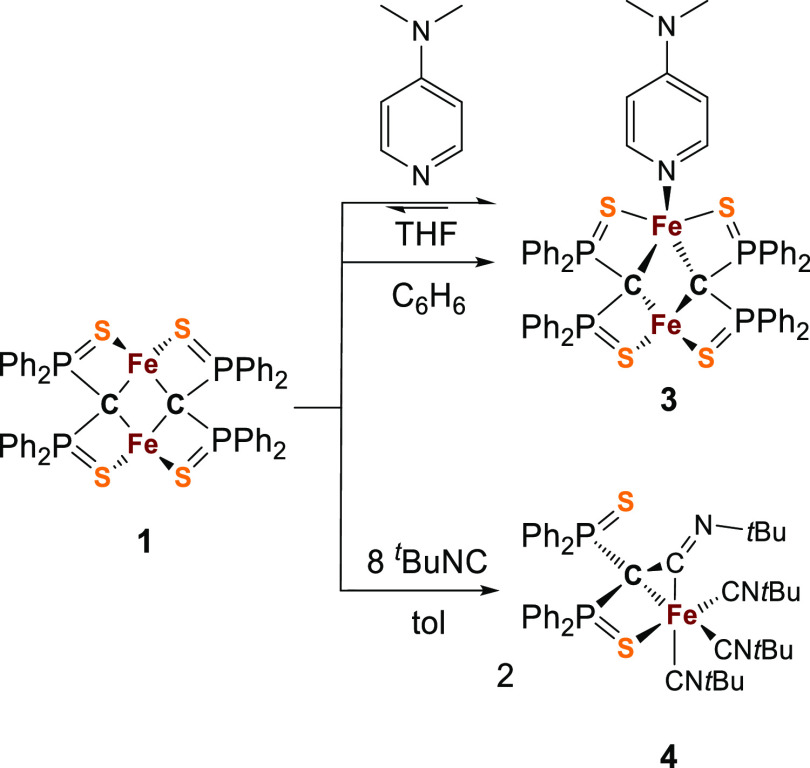
Synthesis of Cluster **3** and Complex **4**

The UV–vis spectrum of **2** displays a characteristic band at 556 nm (Figure S12). The zero-field Mössbauer spectrum recorded at 80 K (Figure 3B) displays two distinct doublets of equal integration with isomer shifts of δ = 0.40 mm s^–1^ (Δ*E*_Q_ = 1.59 mm s^–1^, component 2) for the Fe^III^ site and δ = 0.62 mm s^–1^ (Δ*E*_Q_ = 2.92 mm s^–1^, component 1) for the Fe^II^ site. The difference between the isomer shifts is less than expected from mononuclear complexes with these oxidation states. Further, comparing the Fe^II^ site to that of **1** (δ = 0.70 mm s^–1^ and Δ*E*_Q_ = 3.65 mm s^–1^) implies a degree of valence delocalization between the sites, consistent with a type II Robin–Day classification.^53^ The signals are also observable at 230 K but are slightly shifted to lower values (Fe^III^: δ = 0.35 mm ^s–1^ with Δ*E*_Q_ = 1.55 mm s^–1^; Fe^II^: δ = 0.58 mm s^–1^ with Δ*E*_Q_ = 2.53 mm s^–1^). The reluctancy for the Fe^II^ and Fe^III^ sites to fully delocalize the valence, even at relatively high temperatures, differs between mixed-valent sulfide-bridged clusters: for example, the β-diketiminate-supported Fe^II/III^ complex has distinct Mössbauer signals only at 4.2 K (Fe^III^: δ = 0.47 mm s^–1^ with Δ*E*_Q_ = 1.41 mm s^–1^; Fe^II^: δ = 0.69 mm s^–1^ with Δ*E*_Q_ = 2.90 mm s^–1^), while at 200 K, it exhibits a coalesced doublet (δ = 0.45 mm s^–1^ and Δ*E*_Q_ = 1.41 mm s^–1^).^41^ The bis(benzimidazolato)-stabilized Fe^II/III^ complex similarly displays one doublet at 80 K (δ = 0.50 mm s^–1^ and Δ*E*_Q_ = 0.79 mm s^–1^).^42^ We also performed solid-state SQUID measurements on **2**[BAr_4_^F^], and we fit the data successfully to an *S* = 1/2 ground state. Increasing the temperature from 2 to 270 K results in an increase in μ_eff_ from 2.4 to 3.1 μ_B_, which could be fit as antiferromagnetic coupling with *J* = −118 cm^–1^ [using −2*J*(*S*_1_·*S*_2_)].

**Figure 4:**
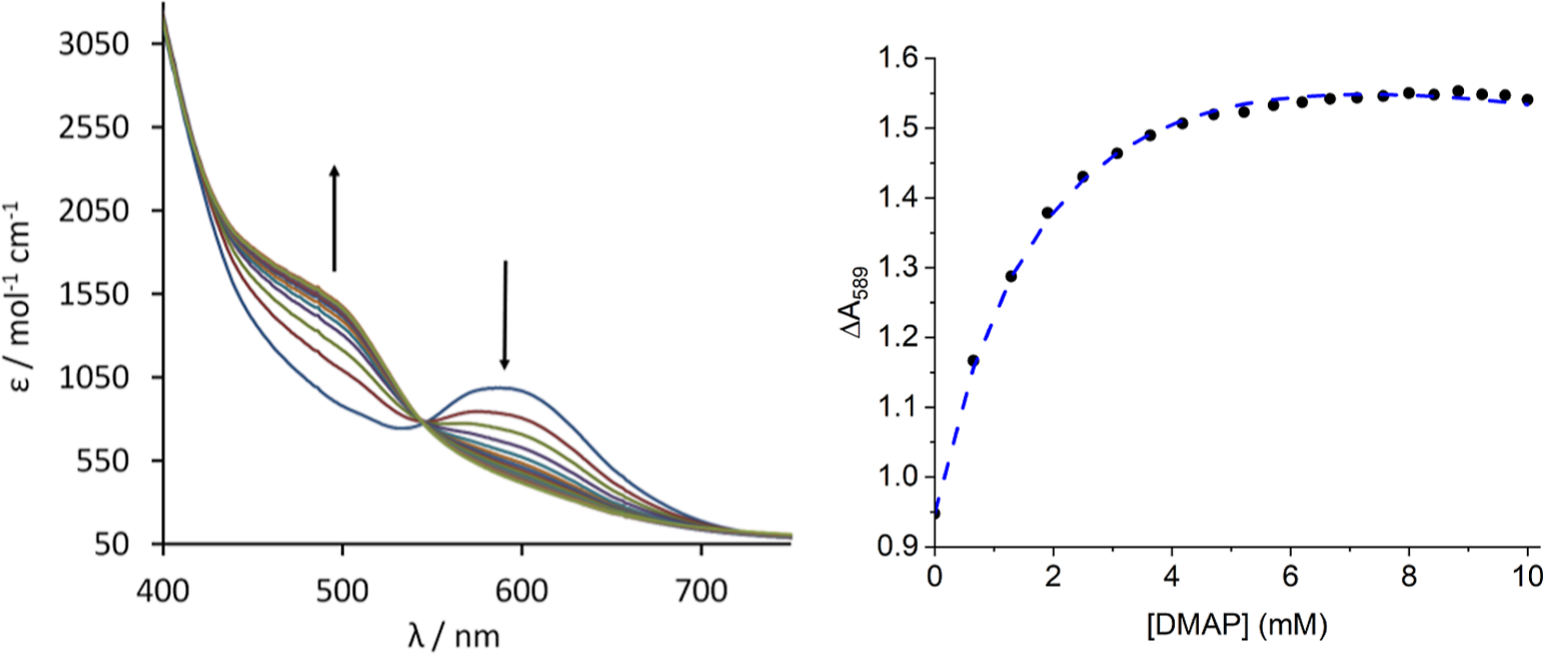
UV–vis spectra (left) and change of the absorbance at 490 nm (right) upon the incremental addition of DMAP to a solution of 1 in THF (0.4 mM); dashed blue curve (right) displays fit by iteration (*K* = 560 ± 10 M^–1^).

In order to understand other parallels of cluster **1** with the belt Fe atoms of the FeMco, we tested whether **1** can bind substrates of the FeMco. However, **1** did not react with the nitrogenase substrates N_2_, CO, or CO_2_ at room temperature.^54^ Nevertheless, the addition of 4-dimethylpyridine (DMAP) resulted in a color change from dark green to red (Scheme 2). Upon the incremental addition of DMAP to a tetrahydrofuran (THF) solution of **1** (0.4 mM), the UV–vis spectra showed a decrease in the distinct absorption band at 589 nm with the growth of a new absorption band at 490 nm (Figure 4, left).

The binding curve derived by monitoring the absorption at 490 nm (Figure 4, right) indicates weak, reversible binding. Using a 1:1 binding model, a binding constant of 560 ± 10 M^–1^ was calculated.^55^ The reversibility of this reaction impedes the isolation of **3** in solvents such as THF, CH_2_Cl_2_, toluene, or fluorobenzene. However, the very low solubility of **3** in benzene enables its isolation upon the addition of DMAP to a saturated solution of **3** in benzene, which causes **3** to precipitate from the reaction mixture, and the solid was amenable to further characterization. Complex **3** is obtained as a deep-red crystalline material in 72% yield and represents a rare example of an iron dimer with mixed CNs. The ^57^Fe Mössbauer spectrum of **3** recorded at 80 K displays two quadrupole doublets, as expected for the inequivalent Fe^II^ sites (Figure 3C). An isomer shift of δ = 0.67 mm s^–1^ with Δ*E*_Q_ = 3.63 mm s^–1^ is assigned to the tetracoordinated Fe^II^ site, based on its similarity to precursor **1**. A higher isomer shift of δ = 0.89 mm s^–1^ with Δ*E*_Q_ = 2.22 mm s^–1^ is assigned to the other Fe^II^ site, and the higher isomer shift is consistent with the increased CN. The solid-state SQUID measurement (2–270 K) reveals a temperature-dependent effective magnetic moment (Figure S16) characteristic of strong antiferromagnetic coupling [*J* = −138 cm^–1^, using −2*J*(*S*_1_·*S*_2_)] between the two high-spin iron(II) sites, resulting in a diamagnetic ground state.

**Figure 5:**
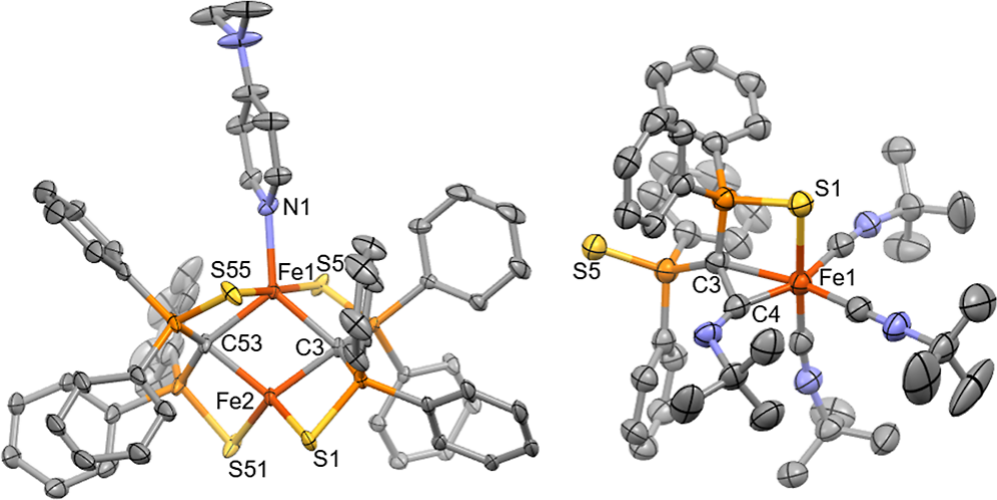
Molecular structure of **3** and **4** (hydrogen atoms are omitted for clarity and thermal ellipsoids are displayed at 50% probability): selected bond lengths in angstroms and angles in degrees for 3: Fe1···Fe2 2.6339(6), Fe1–C3 2.154(2), Fe1–C53 2.148(3), Fe2–C3 2.085(2), Fe2–C53 2.113(3), Fe1–S5 2.5776(8), Fe1–S55, 2.6351(8), Fe2–S1 2.3759(8), Fe2–S51 2.3652(9), Fe1–N 2.100(3), Fe1–C3–Fe2 76.79(8), Fe1–C53–Fe2, 76.35(9), C3–Fe1–C53 101.61(7), C3–Fe2–C53 105.16(10). For 4: Fe1–S1 2.4104(13), Fe1–C3 2.110(4), Fe1–C4 1.899(5), C3–C4 1.508(6), Fe1–C4–C3 75.58(10), C4–C3–Fe1 60.63(10), C3–Fe1–C4 43.75(10), S1–Fe1–C3 79.47(9), Fe1–C3–P1 96.51(8), C3–P1–S1 100.66(9), and P1–S1–Fe1 81.31(8).

The crystallographic structure of **3** reveals a distorted tetrahedral geometry for Fe2 (Figure 5), similar to that of precursor **1**. Fe1, the site of DMAP coordination, has a distorted trigonal bipyramidal geometry (τ_5_ = 0.69, where 1.0 = trigonal bipyramidal and 0.0 = square pyramidal). DMAP coordination results in a slight increase in the Fe···Fe distance to 2.6339(6) Å compared to that in **1** (2.5519(4) Å). The Fe–C bonds about Fe1 are elongated in comparison to that in **1**, with distances of 2.151(5) Å (averaged). Notably, the Fe1–S bond lengths are 2.606(2) Å, significantly elongated as a consequence of the DMAP complexation compared to the Fe2–S bonds [av. 2.341(2) Å]. This is interesting in the context of nitrogenase because the binding of this (admittedly nonbiological) substrate causes a substantial weakening of the Fe–S interactions. Recent mechanistic proposals on the reduction of N_2_ by the FeMoco include cleavage of one of the Fe–S bonds to allow N_2_ to bind.^56,57^ The transformation of **1** to **3** demonstrates that structural reorganization to accommodate an additional ligand is reasonable in a coordination environment similar to that of the FeMco.

**Figure 6:**
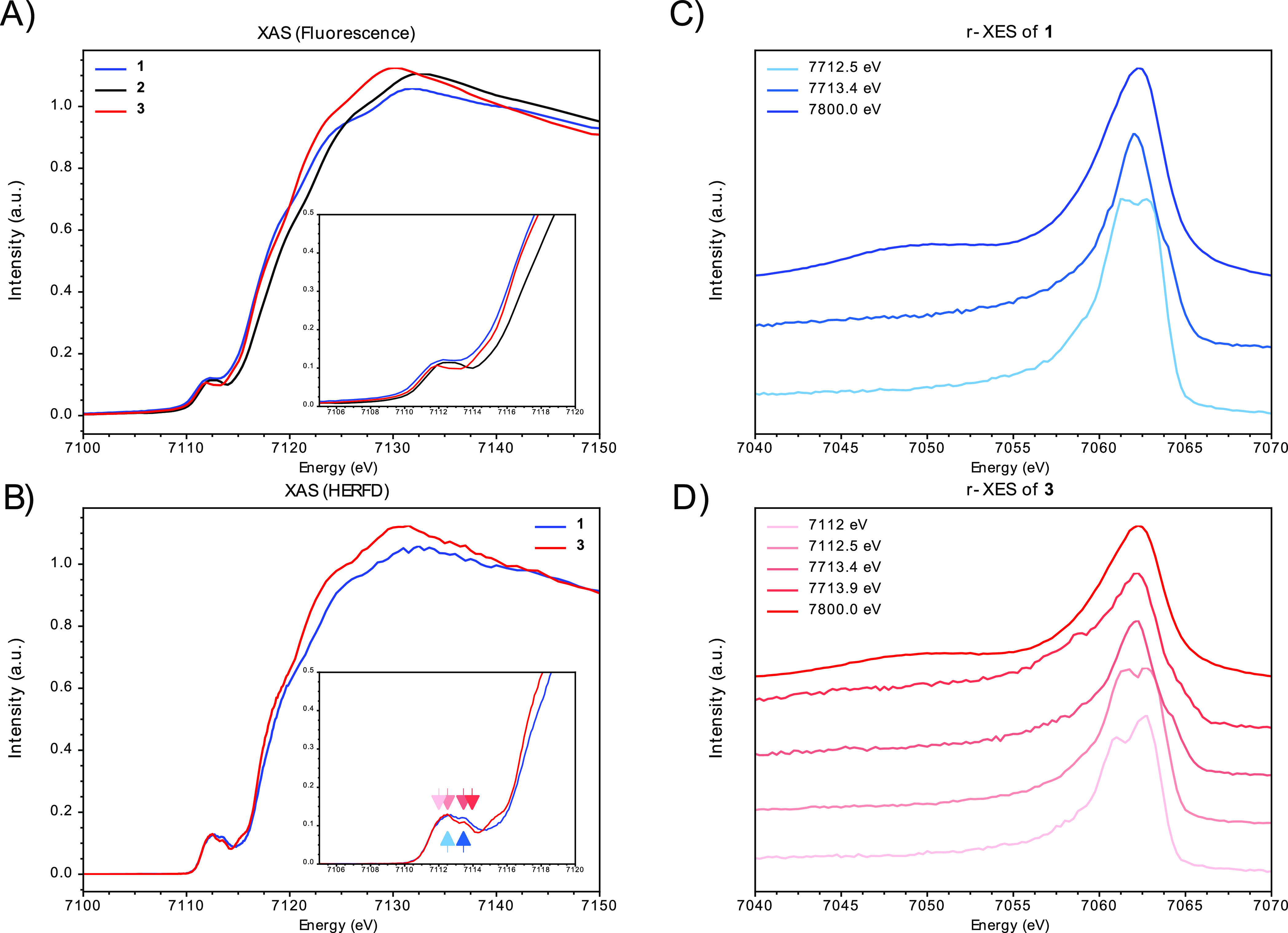
Fe K-edge XAS spectra of complexes **1**, **2**, and **3** (A); Fe Kβ_1,3_ HERFD-XAS spectra of complexes **1** and **3** (B); Fe Kβ_1,3_ RXES of complexes **1** (C) and **3** (D). RXES excitation energies employed in (C) and (D) are indicated by arrows in the pre-edge region inset shown in (B).

The Fe K-edge X-ray absorption spectroscopy (XAS) spectra of complexes 1, 2, and 3 are presented in Figure 6. The rising edge shifts to higher energy by 0.7 eV moving from the di-iron(II) complexes **1** and **3** to the mixed-valence complex **2**, consistent with oxidation.^58^ A comparison of the first derivatives of the XAS spectra is provided in the Supporting Information (Figure S17). These data support the assignment of all-ferrous oxidation levels in 1 and 3. Fe Kβ_1,3_ high-energy-resolution fluorescence detected XAS (HERFD-XAS) spectra of **1** and **3** each exhibit two pre-edge features at 7112.4 and 7113.5 eV, with the latter exhibiting decreased intensity in **3**. Additionally, compound **3** displays a low-energy rising edge feature at 7115.2 eV that is not observed in **1**, which may arise from a metal-to-metal charge transfer transition as previously observed in both the MoFe- and Mo-based heterocubanes.^46^ The iron(II) oxidation levels in **1** and **3** are also confirmed by resonant X-ray emission Kβ_1,3_ spectroscopy (RXES) as shown in Figure 6C,D, where characteristic double peaks are observed when using incident energies corresponding to the pre-edge region. This double peak arises from the differences in the final-state configurations (5D, 5F, and 5G) of the 1s 3p resonant and nonresonant processes and has previously been observed for L_2_Fe^II^Fe^II^S complexes with β-diketiminate-supporting ligands (L1-).^59^ Under nonresonant conditions, the Kβ_1,3_ mainlines of **1** and **3** are identically shaped (Figure S18); however, subtle differences are observed under resonant conditions (Figure 6C,D), which are consistent with modulations in the ligand field parameters of **3** relative to those of **1**.^58,60^ Unfortunately, differentiating the contributions of the two iron sites in **3** is beyond the resolution of the current experiments.

Methyl isocyanide is a substrate for nitrogenase, being reduced to methane and methylamine in a six-electron process or dimethylamine in a four-electron process.^61,62^ Further, isocyanides share the steric profile of N_2_ and have comparable π-accepting properties, while being significantly stronger electron σ-donors. The addition of 1 equiv of *tert*-butyl isocyanide to **1** resulted in partial conversion to a new species, as observed by ^1^H and ^31^P NMR spectroscopy. A further 7 equiv was required in order to reach full conversion to a single species (Scheme 2). The ^1^H NMR spectrum features four resonances in the region of isocyanide ^*t*^Bu groups, while the ^31^P NMR spectra display an AX spin system with resonances of equal intensity at 55.9 and 44.2 ppm (^2^*J*_P–P_ = 35 Hz), indicative of the inequivalent phosphorus environments. Crystals were grown from saturated Et_2_O solution in 44% yield revealing **4** to be a monometallic Fe complex in which one of the S ligands has been displaced from the metal and a new C–C bond has formed between the isocyanide C atom and C of the yldiide (Figure 5). Thus, this donor goes beyond weakening the Fe–S bond to completely breaking it, which may be relevant to current proposals of the nitrogenase mechanism in which Fe–S cleavage is crucial - though some of the structural support for this idea is under debate.^63−67^ The insertion of isocyanide into a Fe–C bonds is frequently encountered in organometallic chemistry.^68,69^ The Mössbauer spectrum of **4** (Figure 3D) displays a doublet (δ = 0.12 mm s^–1^ and Δ*E*_Q_ = 1.89 mm s^–1^), which is comparable to that of previously reported octahedral, low-spin Fe^II^ complexes.^70,71^

## Conclusions

Sulfur-based yldiide-bridged ligands are shown to be useful tools for preparing iron complexes with sulfur–carbon environments. We found that oxidizing Fe^II^ dimer **1** yielded a mixed-valent Fe^II/III^ dimer, **2**, and its coordination environment has some similarities to the iron sites in the catalytic nitrogenase cofactors. Mössbauer spectroscopy revealed **2** to have localized Fe^II^ and Fe^III^ sites up to 230 K, indicating that the yldiide-bridging ligands result in slower electron transfer between the two sites than sulfide-bridged dimers. Even though these compounds did not bind weak ligands like N_2_, we found that **1** does bind 4-dimethylaminopyridine (DMAP) to give an asymmetric Fe^II^ dimer, with DMAP bound to a 5-coordinate Fe site. An isocyanide, on the other hand, cleaved the dimer to form a monomeric complex featuring a new C–C bond between the ligand backbone and the C atom of the isocyanide. This reactivity of the carbon group of the supporting ligand may limit the utility of this ligand for nitrogenase modeling.

## Supporting Information Available

Synthetic procedures for all complexes; ^1^H NMR spectra of **1**, **2**, and **4**; FT-IR and UV spectra of metal complexes; cyclic voltammetry of **1**; Mössbauer spectra and assignments; magnetic measurements of **1**, **2**, and **3**; crystallographic data for **1**, **3**, and **4**; and XAS and X-ray emission spectroscopy measurements; and references (PDF)
