# Supplementary material for: Sulfur-Ligated [2Fe-2C] Clusters as Synthetic Model Systems for Nitrogenase
Source: Inorg Chem. 2023 Jan 30;62(6):2663–71. doi: 10.1021/acs.inorgchem.2c03693 (PMC9930126; doi:10.1021/acs.inorgchem.2c03693)
Supplement: Supplementary file 1 — ic2c03693_si_001.pdf [file ic2c03693_si_001.pdf]

Supplementary Information

Sulfur-Ligated [2Fe-2C]-Clusters as Synthetic Model Systems for Nitrogenase

Sivathmeehan Yogendra†, Daniel W. N. Wilson‡, Anselm W. Hahn†, Thomas Weyhermüller†, Casey Van Stappen†, Patrick Holland‡,*, Serena DeBeer†,*

† Max Planck Institute for Chemical Energy Conversion, Stiftstrasse 34-36, 45470 Mülheim an der Ruhr, Germany

‡ Department of Chemistry, Yale University, 225 Prospect St., New Haven, Connecticut 06520, USA

Serena.Debeer@cec.mpg.de

Patrick.Holland@yale.edu

Contents

General considerations....2
Synthetic procedures....3
Modified synthesis of $[LFe]_2$, **1**[4]....3
Synthesis of $[LFe]_2[BArF]$, **2**....4
Synthesis of [LFe(DMAP)FeL], **3**....5
Synthesis of **4**....6
^{1}H NMR spectra of **1**, **2**, and **4**....7
FTIR spectra of metal complexes....10
UV-vis spectra....11
Cyclic voltammetry....14
Mössbauer spectra and assignments....15
Magnetic measurements....16
Crystallographic data....19
X-ray Absorption (XAS) and Emission (XES) Spectroscopy Methods....21
References....25

General considerations

All manipulations were performed in an argon-filled MBraun glovebox maintained below 1 ppm of O_2 and H_2O or under an N_2 atmosphere using standard Schlenk techniques unless mentioned otherwise. Glassware was oven-dried at 150 °C for at least 12 h prior to use. Celite and molecular sieves were dried above 200 °C under vacuum for at least 12 h. Pentane, THF, hexanes, benzene, toluene, and diethyl ether were purified by passage through activated alumina and Q5 columns from Glass Contour Co, under argon, and stored over activated molecular sieves. Benzene-d_6 and THF-d_8 were vacuum transferred from a solution of potassium benzophenone ketyl and was stored over 4 Å molecular sieves. LLi_2,[1] Ferrocenium BAr^F ($Fc[\{3,5-(CF_3)_2C_6H_3\}_4B]$),[2] and $FeCl_2(THF)_{1.5}$[3] were prepared by literature procedures. *tert*-Butyl isocyanide was purchased from Sigma Aldrich, degassed, and stored over molecular sieves prior to use. 4-Dimethylaminopyridine (DMAP) was purchased from ThermoFisher and used as received.

NMR data were collected on Agilent 400 or 500 MHz spectrometers. Chemical shifts in ^1H NMR spectra are referenced to the residual proton solvent peaks of C_6D_5H (δ 7.16 ppm), THF-d_8 (δ 3.58 ppm), and $CDHCl_2$ (5.32 ppm). Elemental analyses were performed at the CENTC Elemental Analysis Facility at the University of Rochester. IR spectra were collected on an Alpha Platinum ATR IR Spectrometer. UV-vis spectra were recorded on a Cary 50 spectrometer using Schlenk-adapted quartz cuvettes with a 1, 2, or 10 mm path length.

Synthetic procedures

Modified synthesis of $[LFe]_2$, 1[4]

Crystals of LLi_2 (250 mg, 0.543 mmol) were dissolved in THF (5 mL) and cooled to –78 °C. Solid $FeCl_2(THF)_{1.5}$ (128 mg, 0.543 mmol) was suspended in THF and added dropwise to the solution of LLi_2. The remaining $FeCl_2(THF)_{1.5}$ was rinsed from the vial with THF (3 × 1 mL) and added to the reaction mixture. After a few minutes of stirring, the reaction turned dark brown. The reaction mixture was allowed to warm to room temperature and stirred overnight. The solvent was removed, and the resulting solids were taken into benzene (30 mL) and filtered through a medium porosity glass frit. The remaining solids were washed with benzene (3 mL portions) until the filtrate ran colorless. The filtrate was then dried under vacuum and washed with THF (2 × 5 mL), Et_2O (2 × 5 mL), and hexane (2 × 5 mL) and dried under vacuum to give **1** as a green powder. Analytically pure crystals of **1** were grown by layering saturated MeCN solution with Et_2O at –35 °C (215 mg, 39%).

^{1}H NMR (400 MHz, CD_2Cl_2) δ (ppm) 10.88 (4H), 8.37 (br, 3H), 7.63 (4H), 6.85 (2H), 4.81 (2H), 1.28 (0.5H, impurity), 0.88 (0.5H, impurity). **^{1}H NMR** (400 MHz, C_6D_6) δ (ppm) 10.12 (4H), 8.51 (br, 3H), 7.06 (4H), 6.28 (2H), 4.83 (br, 2-3H), 3.94 (2H). **UV-vis** (benzene, ε in mM^{-1} cm^{-1}) 590 nm. **Mössbauer:** δ = 0.70 mm s^{-1}, ΔE_Q = 3.65 mm s^{-1}, FWHM 0.30 mm s^{-1}. **IR** (ATR, neat. cm^{-1}): 3590, 3055, 2925, 2119, 1980, 1813, 1585, 1574, 1480, 1435, 1309, 1183, 1161, 1095, 1068, 1027, 998, 832, 772, 740, 723, 685, 664, 595, 578, 556. **Elemental analysis** calculated for $C_{50}H_{40}Fe_2P_4S_4$: C, 59.77; H, 4.01; N, 0.00. Found: C, 59.12; H, 4.21; N, 0.09.

Synthesis of $[LFe]_2[BArF]$, 2

Separately, $[LFe]_2$ (52 mg, 0.052 mmol) and $[Cp_2Fe][BArF]$ (58 mg, 0.052 mmol) were dissolved in THF (2 mL) and both solutions were cooled to –78 °C for 20 minutes. The solution of $[Cp_2Fe][BArF]$ was added dropwise to $[LFe]_2$. Once the addition was complete, the reaction mixture was allowed to warm to room temperature and stirred for 15 minutes. The resulting red solution was filtered through a medium porosity frit and the filtrate was isolated. Removal of the solvent resulted in a brick red solid, which was washed with Et_2O (3 × 2 mL) and pentane (1 × 2 mL) to yield 2 (75 mg, 77%). Attempts at crystallization failed due to the instability of this complex in solution and the solid state, resulting in the formation of **1** and other unidentified species over time.

^{1}H NMR (400 MHz, CD_2Cl_2) δ (ppm) 10.04 (d, J = 7.6 Hz, 1H), 8.93 (1H), 8.69 (d, J = 7.6 Hz, 1H), 8.15 (1H), 7.72 (3H), 7.53 (2H), 5.16 (br, 3H). **^{1}H NMR** (400 MHz, C_6D_6) δ (ppm) 9.43 (2H), 8.81 (2H), 8.45 (4H), 8.21 (4H), 7.69 (2H), 6.91 (2H), 4.90 (Et_2O), 3.99 (Fc), 3.29 (Et_2O), 1.68 (4H), 1.12 (impurity). **Mössbauer**: δ = 0.70 mm s^{-1}, ΔE_Q = 3.65 mm s-1, FWHM 0.30 mm s^{-1}. **IR** (ATR, neat. cm^{-1}): 3061, 2193, 2077, 1967, 1610, 1587, 1480, 1437, 1353, 1273, 1117, 1094, 1028, 998, 886, 839, 770, 723, 711, 681, 668, 576, 527, 495, 449. UV-vis (benzene, ε in mM^{-1} cm^{-1}) 485 nm. **Elemental analysis**: calculated for $C_{82}H_{52}BF_{24}Fe_2P_4S_4$: C, 52.73; H, 2.81; N, 0.00. Found: C, 53.11; H, 2.99; N, 0.12.

Synthesis of [LFe(DMAP)FeL], 3

A solution of 4-dimethylaminopyridine (14.5 mg, 0.118 mmol) in benzene (0.5 mL) was quickly added to a saturated solution of $[LFe]_2$ (70 mg, 0.07 mmol) in benzene (18 mL) resulting in an immediate color change from green to red. The reaction mixture was shaken for five seconds and was stored for 24 h at ambient temperature resulting in the formation of some red crystals. n-hexane (2 mL) was added and the reaction mixture was held for 48 h to give additional red crystals. The crystalline material was separated by decantation, washed with n-hexane (3 × 0.5 mL), and dried in vacuo to give analytically pure, air-sensitive red crystals (57 mg, 72%).

mp.: decomp. >196 °C. **IR** (ATR, 298 K, in cm^{-1}): 403 (vs), 409 (vs), 416 (vs), 422 (vs), 407 (vs), 421 (vs), 493 (vs), 526 (vs), 556 (vs), 578 (vs), 595 (vs), 664 (vs), 685 (vs), 723 (vs), 740 (vs), 772 (vs), 832 (vs), 998 (vs), 1027 (vs), 1068 (vs), 1096 (vs), 1161 (vs), 1183 (vs), 1309 (vs), 1435 (vs), 1480 (vs), 1574 (vs), 1585 (vs), 2925 (vs), 3055 (vs), 3590 (vw). **Mössbauer** (80 K, 0.0 T): 0.67 mms^{-1} (ΔE_Q = 3.63 mm s^{-1}, rel. int. = 48%), 0.89 mms^{-1} (ΔE_Q = 2.22 mms^{-1}, rel. int. = 52%). **UV/Vis** (THF, λ_{max} in nm, ε_M in M^{-1} cm^{-1}): 490 (1548). **Elemental analysis**: calculated for $C_{57}H_{50}Fe_2N_2P_4S_4$: C, 60.75; H, 4.47; N, 2.49. **Found**: C, 60.71; H, 4.66; N, 2.48.

Synthesis of 4

To a stirring solution of **1** (118 mg, 0.118 mmol) in toluene (3 mL) was added a solution of ^{t}BuNC (80 mg, 0.962 mmol) in toluene (1 mL). The solution immediately turned from green to red. The solvent was removed, and the resulting red oil was washed with hexane (3 × 2 mL) to leave an orange solid. The remaining solid was taken into THF (4 mL) and filtered. The dark yellow solution was concentrated to 1 mL and 10 drops of hexane were added. Cooling this solution to –35 °C overnight yielded yellow crystals of **4** (86 mg, 44%).

^{1}H NMR (400 MHz, C_6D_6) δ 8.82 (dd, $^3J_{H–H}$ = 13.6, 7.6 Hz, 2H, *ortho*-ArC*H*), 8.68 (dd, $^3J_{H–H}$ = 13.6, 7.6 Hz, 2H, *ortho*-ArC*H*), 8.41 (dt, $^3J_{H–H}$ = 7.1, 12.5 Hz, 4H, *meta*- and *para*-ArC*H*), 7.89 (dd, $^3J_{H–H}$ = 12.5, 7.1 Hz, 0.4 H, protic ligand impurity), 7.14–6.79 (m, 12H, ArC*H*), 1.85 (s, 9H, ^{t}Bu-CH_3), 1.40 (s, 9H, ^{t}Bu-CH_3), 1.14 (s, 9H, ^{t}Bu-CH_3), 0.89 (s, 9H, ^{t}Bu-CH_3). **^{13}C NMR** (151 MHz, C_6D_6) δ 211.09 (t, $^2J_{C–P}$ = 5.5 Hz, bridging ^{t}BuN*C*), 176.84 (s, ^{t}BuN*C*), 176.21 (s, ^{t}BuN*C*), 170.97 (s, ^{t}BuN*C*), 142.74 (d, $J_{C–P}$ = 59.2 Hz, Ar*C*H), 138.34 (dd, $^1J_{C–P}$ = 82.1, 5.3 Hz, P*C*(Fe)P), 136.85–136.04 (m, Ar*C*H), 135.18 (dd, $J_{C–P}$ = 10.5, 6.5 Hz, Ar*C*H), 132.77 (d, $J_{C–P}$ = 11.0 Hz, Ar*C*H), 132.31–132.00 (m, Ar*C*H), 130.73 (d, $J_{C–P}$ = 125.0 Hz, Ar*C*H), 129.24, 127.48 (d, $J_{C–P}$ = 12.0 Hz, Ar*C*H), 127.09 (d, $J_{C–P}$ = 12.5 Hz, Ar*C*H), 126.65 (dd, $J_{C–P}$ = 19.6, 12.0 Hz, Ar*C*H), 57.15 (s, CN*C*$(CH_3)_3$), 55.67(s, CN*C*$(CH_3)_3$), 55.58 (s, CN*C*$(CH_3)_3$), 55.44 (s, CN*C*$(CH_3)_3$), 31.41(s, CNC$(CH_3)_3$), 31.36 (s, CNC$(CH_3)_3$), 31.32 (s, CNC$(CH_3)_3$), 31.09 (s, CNC$(CH_3)_3$). **^{31}P{^{1}H} NMR** (162 MHz, C_6D_6) δ 55.92 (d, $^2J_{P–P}$ = 34.5 Hz), 44.15 (d, $^2J_{P–P}$ = 34.5 Hz). **IR** (ATR, neat. cm^{-1}): 3050, 2973, 2116 (asymmetric RNC), 2086 (shoulder, asymmetric RNC), 2039 (asymmetric RNC), 1662 (symmetric RNC, or N=C stretch), 1606 (symmetric RNC, or N=C stretch),1432, 1365, 1355, 1229, 1201, 1100, 1026, 991, 872, 774, 739, 711, 687. **Elemental analysis** calcd for $C_{45}H_{56}FeN_4P_2S_2$: C, 64.74; H, 6.76; N, 6.71. **Found:** C, 64.85; H, 6.82; N, 6.56.

^{1}H NMR spectra of 1, 2, and 4

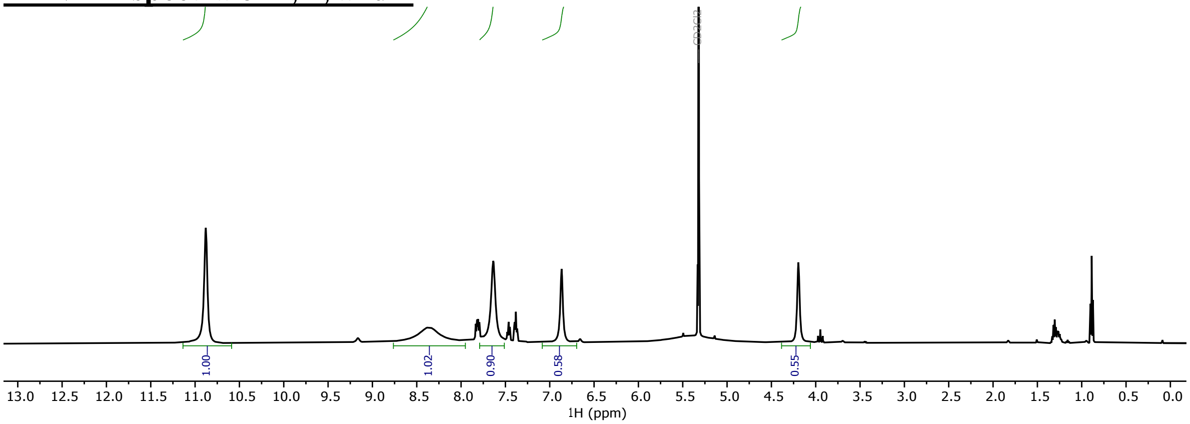

Figure S 1. ^{1}H NMR (400 MHz) spectrum of **1** in CD_2Cl_2.

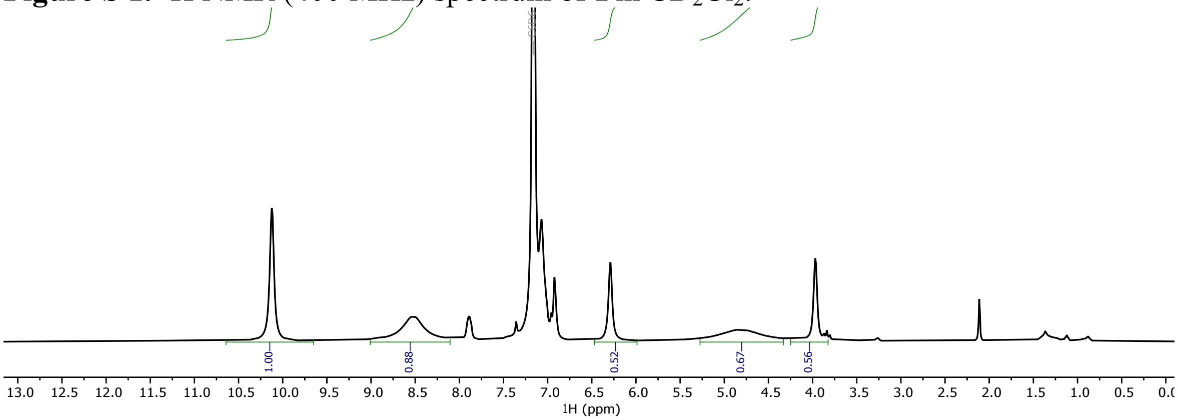

Figure S 2. ^{1}H NMR (400 MHz) spectrum of **1** in C_6D_6.

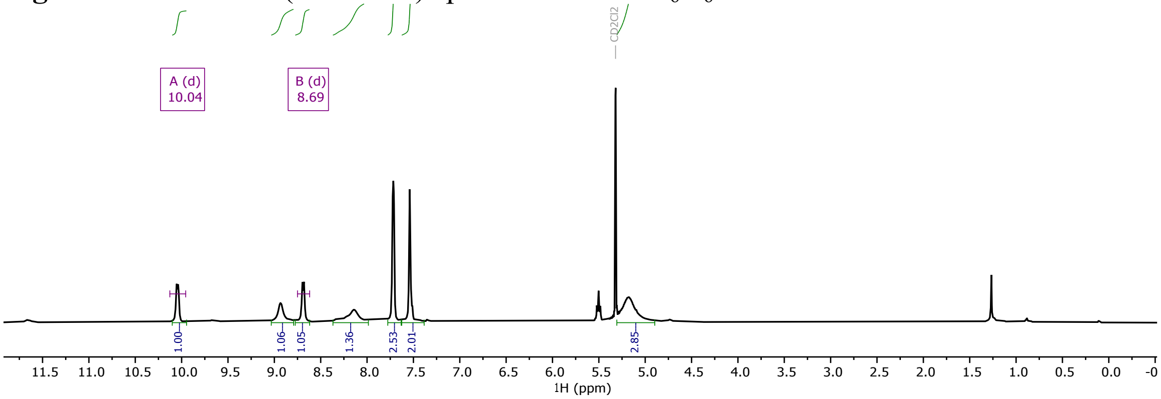

Figure S 3. ^{1}H NMR (400 MHz) spectrum of **2** in CD_2Cl_2.

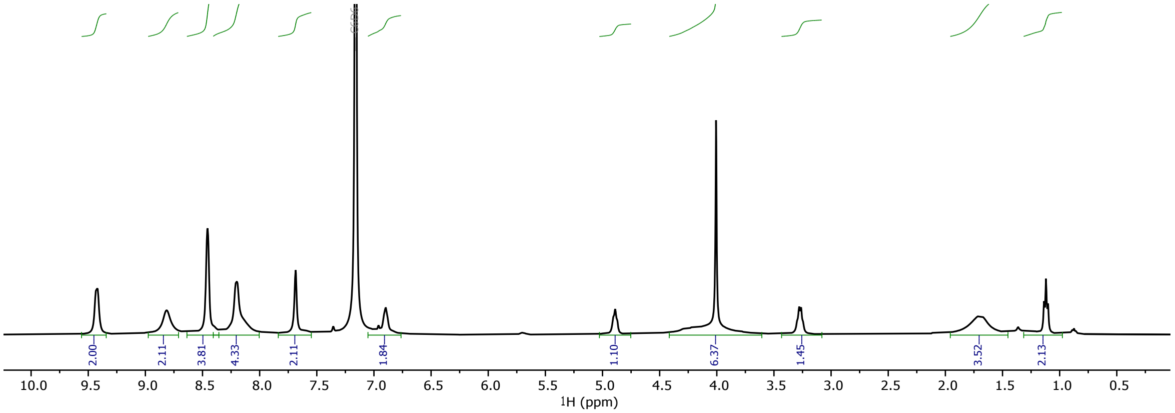

Figure S 4. ^{1}H NMR (400 MHz) spectrum of **2** in C_6D_6.

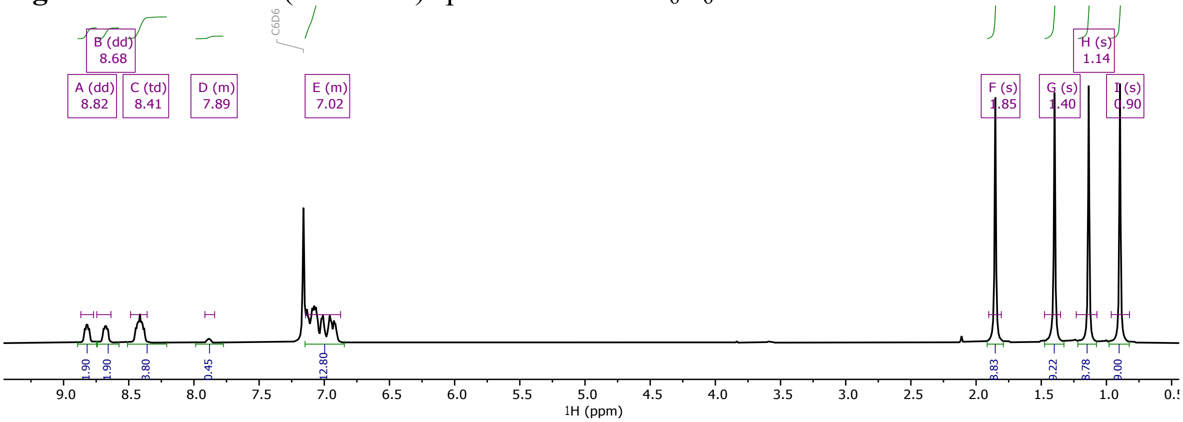

Figure S 5. ^{1}H NMR (400 MHz) spectrum of **4** in C_6D_6.

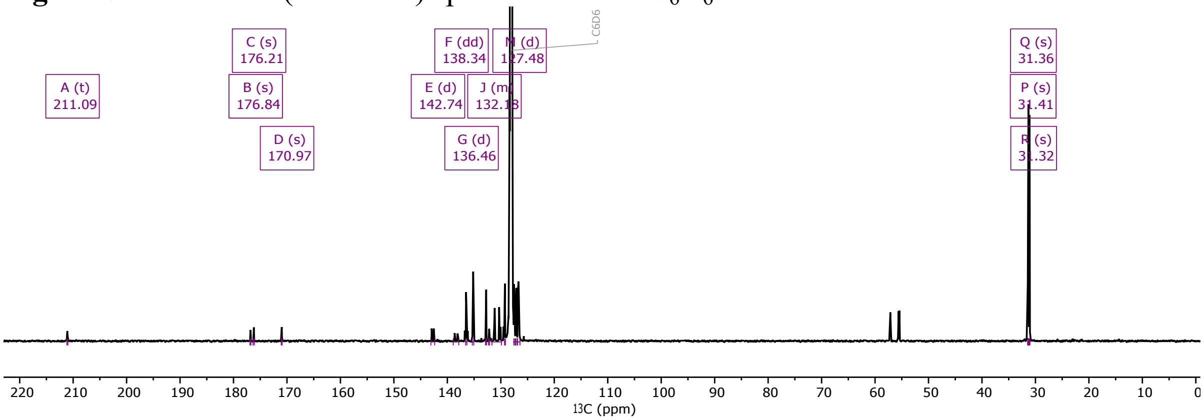

Figure S 6. ^{13}C NMR (151 MHz) spectrum of **4** in C_6D_6.

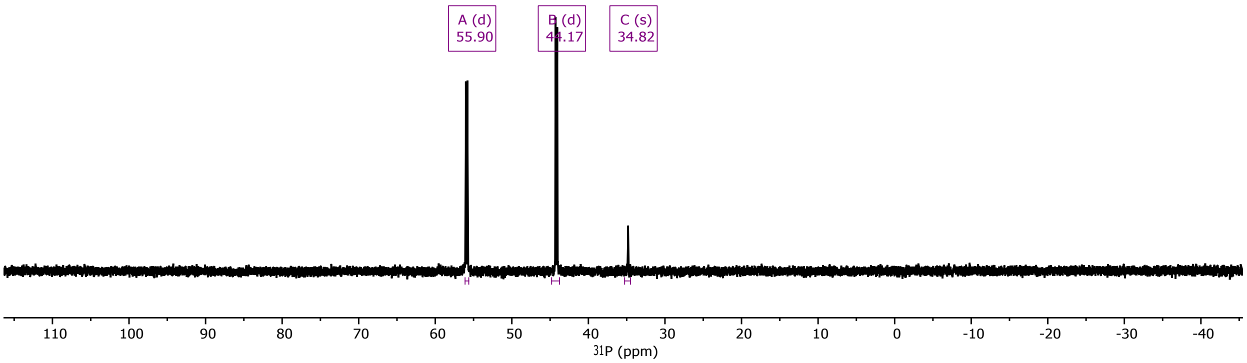

Figure S 7. $^{31}P\{^1H\}$ NMR (128 MHz) spectrum of **4** in C_6D_6. Resonance at 34.8 ppm is the free, protonated ligand (<3 % by integration)

FTIR spectra of metal complexes

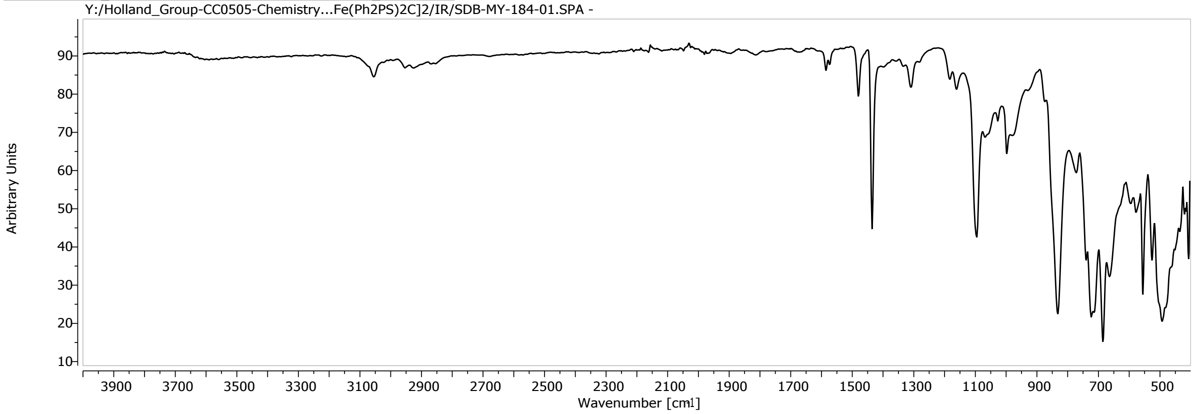

Figure S 8. FTIR spectrum of **1**.

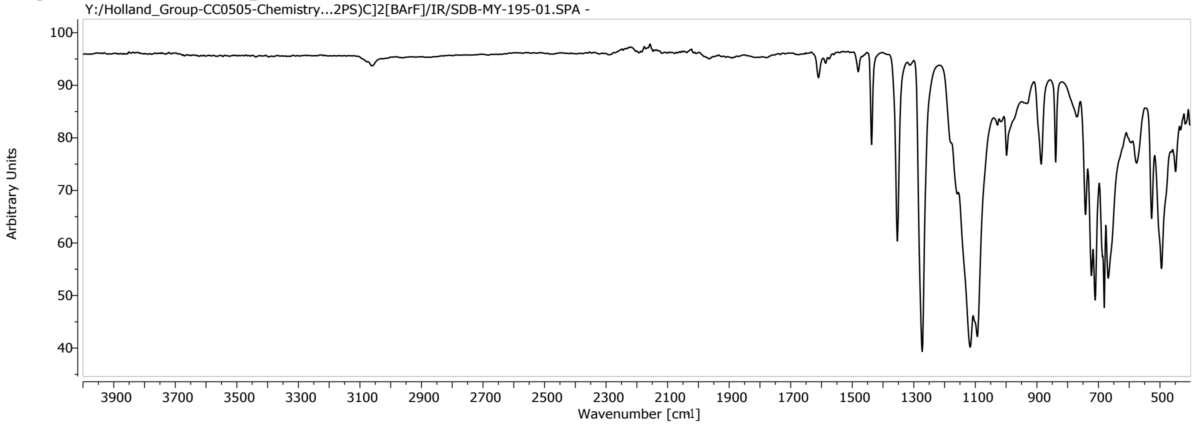

Figure S 9. FTIR spectrum of **2.**

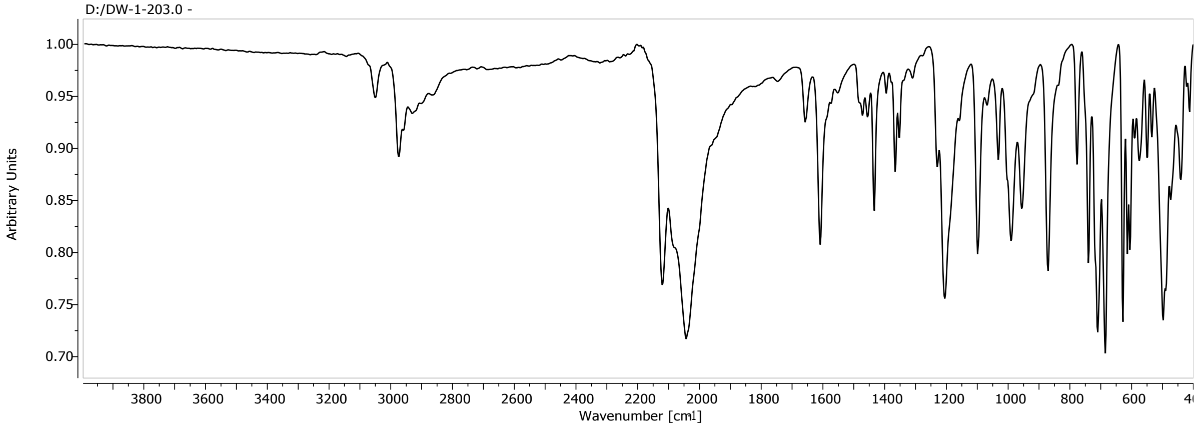

Figure S 10. FTIR spectrum of **4**.

UV-vis spectra

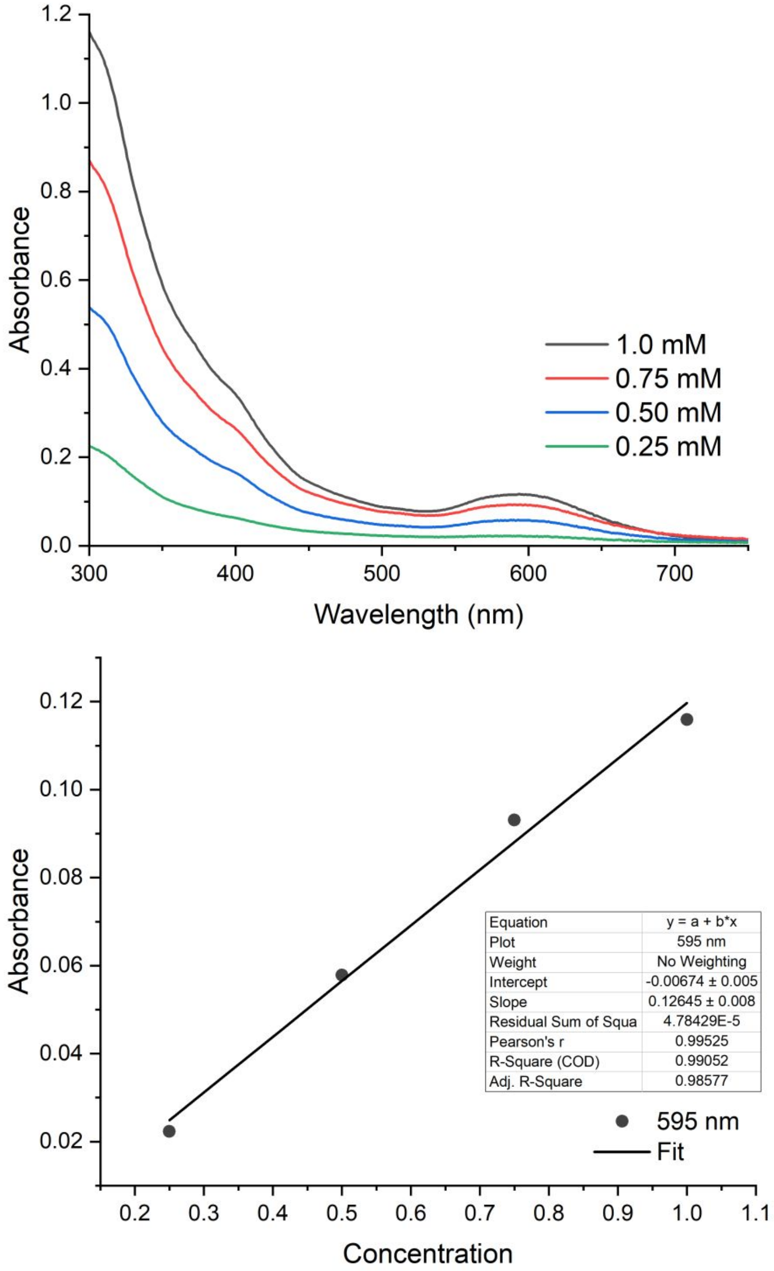

Figure S 11. Top: UV-vis spectrum of **1** in 0.25-1.0 mM solutions in toluene. Bottom: Beer-Lambert Law plot of absorbance at 595 nm in toluene. Trendline equations are in the insets.

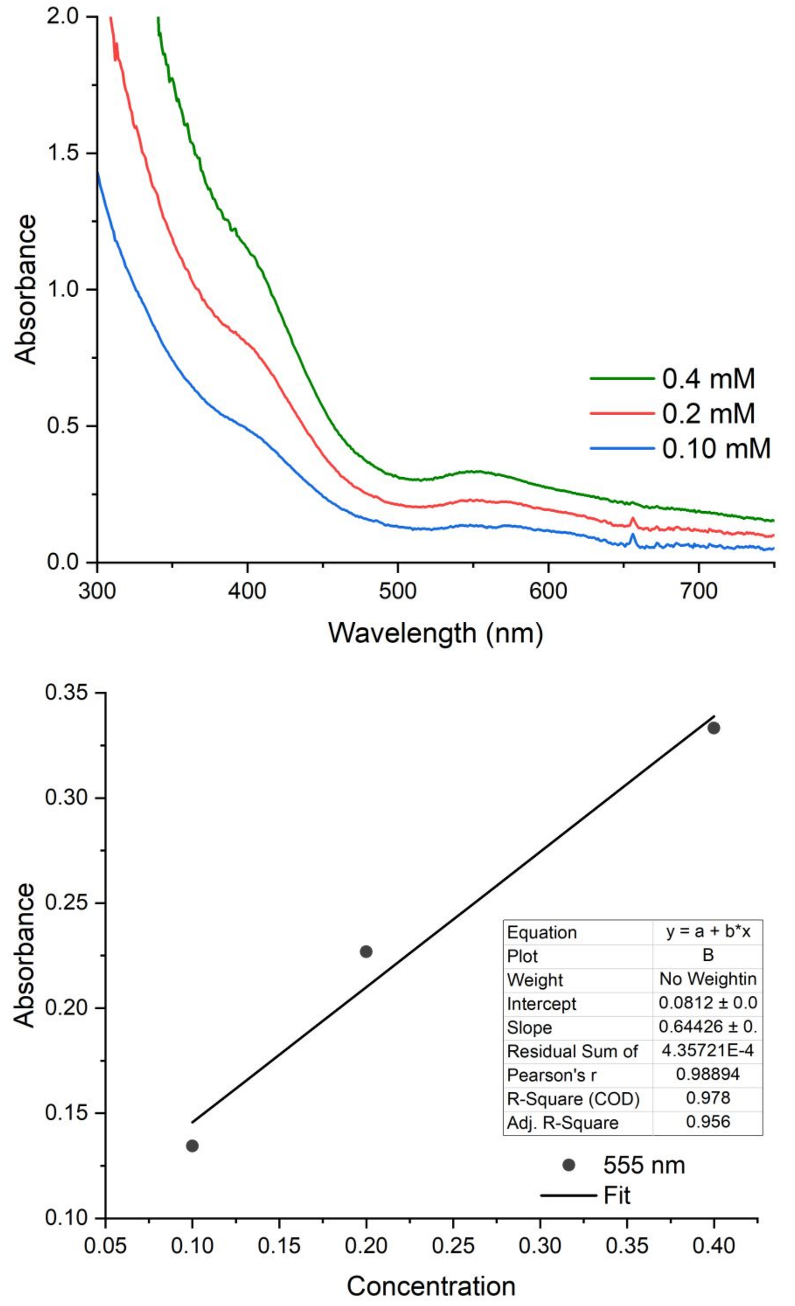

Figure S 12. Top: UV-vis spectrum of **2** in 0.1-0.4 mM solutions in toluene. Bottom: Beer-Lambert Law plot of absorbance at 555 nm in toluene. Trendline equations are in the insets.

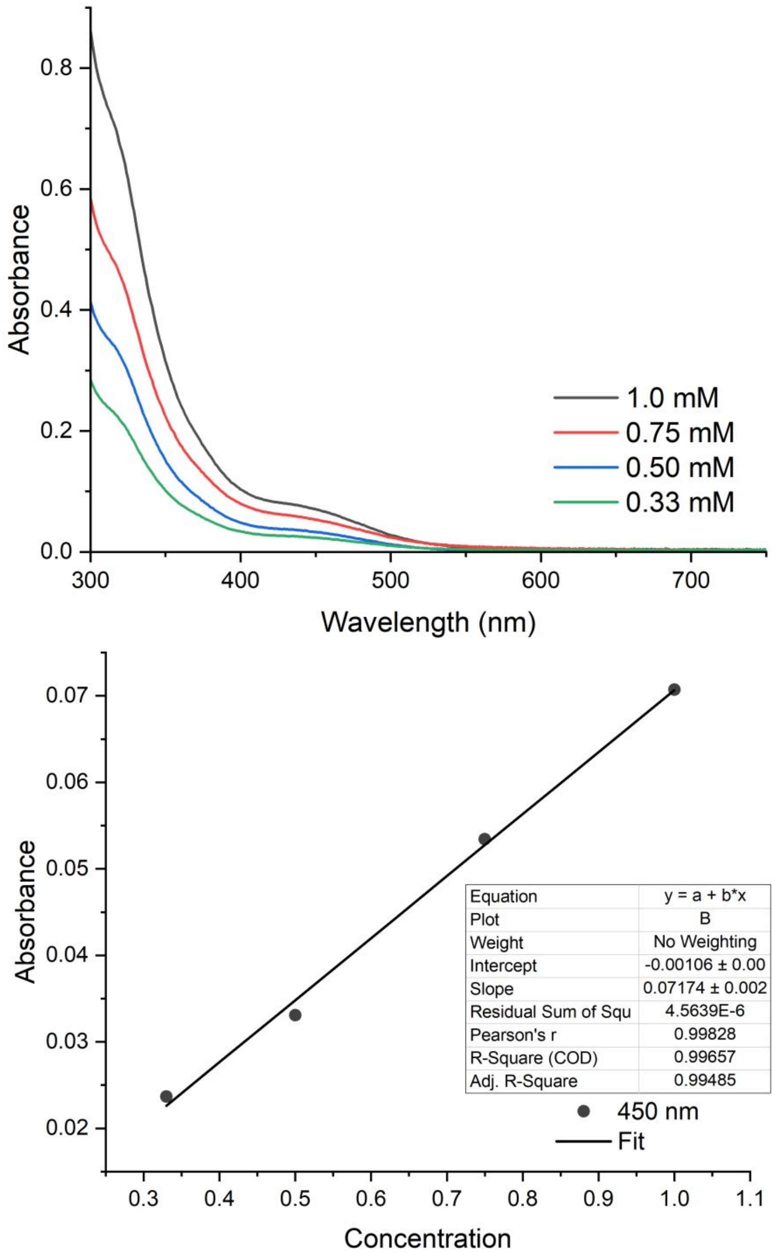

Figure S 13. Top: UV-vis spectrum of **4** in 0.33-1.0 mM solutions in toluene. Bottom: Beer-Lambert Law plot of absorbance at 450 nm in toluene. Trendline equations are in the insets.

Cyclic voltammetry

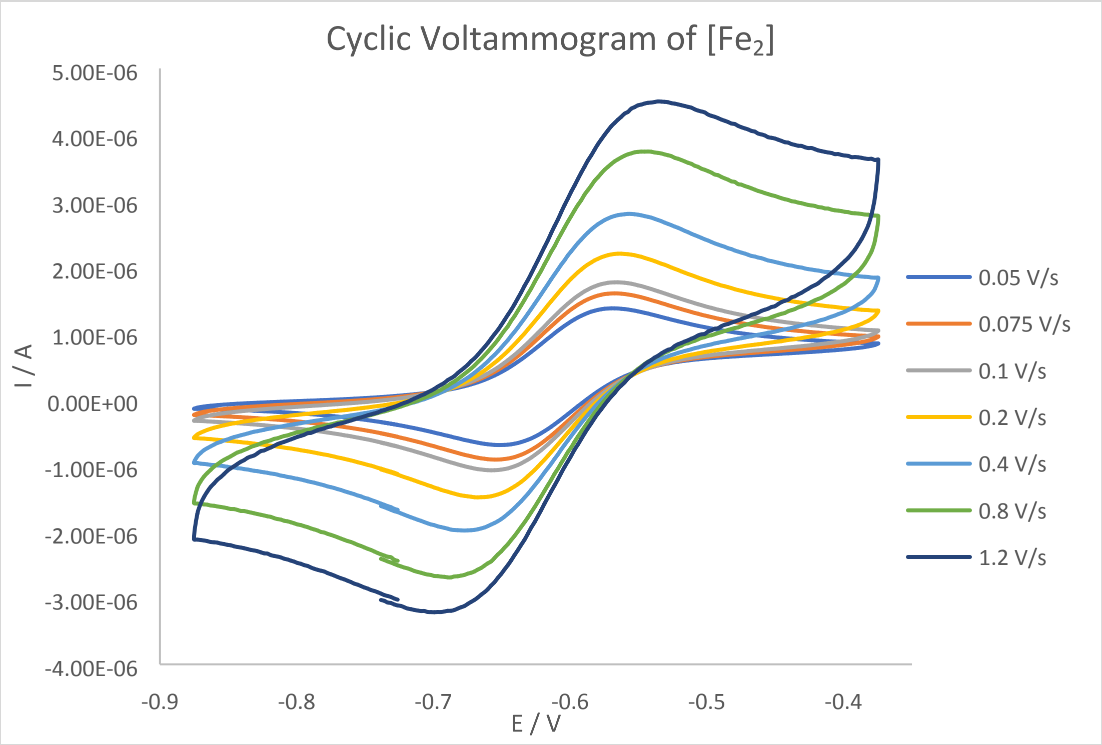

Figure S 14. Cyclic voltammogram of **1/2** redox couple in CH_2Cl_2 at 255 K using $[Bu_4N]PF_6$ as the electrolyte referenced to ferrocene.

Mössbauer spectra and assignments

Mössbauer spectra were recorded on a conventional spectrometer with an alternating constant acceleration of the γ-source. The minimum experimental line width was 0.24 mm/s (full width at half height). The sample temperature was maintained constant in an Oxford Instruments Variox cryostat. The ^{57}Co/Rh source (0.6 GBq) was kept at room temperature. Isomer shifts are quoted relative to iron metal at 300 K. Zero field spectra were measured at 80 K.

Table S 1. Zero-field Mössbauer fitting parameters for **1-4** at 80 K.

Complex	Isomer shift (δ)	Quadrupole Splitting (ΔE_Q)	$\Gamma_{L/R}$ FWHM	Relative Area
**1**	0.70 mm s^{-1}	3.65 mm s^{-1}	0.30 mm s^{-1}	100%
**2**	0.40 mm s^{-1}<br>0.62 mm s^{-1}	1.59 mm s^{-1}<br>2.92 mm s^{-1}	1.27 mm s^{-1}<br>1.06 mm s^{-1}	50%<br>50%
**3**	0.67 mm s^{-1}<br>0.89 mm s^{-1}	3.63 mm s^{-1}<br>2.22 mm s^{-1}	0.29 mm s^{-1}<br>0.30 mm s^{-1}	48%<br>52%
**4**	0.12 mm s^{-1}	1.98 mm s^{-1}	0.34 mm s^{-1}	100%

Magnetic measurements

Magnetic susceptibility data were measured from powder samples of solid material in the temperature range 2–290 K by using a SQUID magnetometer with a field of 1.0 T (MPMS-7, Quantum Design, calibrated with a standard palladium reference sample, error < 2%). Sample holders of quartz with an O-ring sealing were used. The SQUID response curves (raw data) have been corrected for holder and solvent contributions by subtracting the corresponding response curves obtained from separate measurements without sample material. In addition, the experimental magnetization data obtained from independent simulation of the corrected SQUID response curves were corrected for underlying diamagnetism by use of tabulated Pascal's constants, as well as for temperature-independent paramagnetism (TIP).[5] Magnetic susceptibility data were analyzed and simulated using the julX software developed by E. Bill (Max Planck Institute for Chemical Energy Conversion, Mülheim an der Ruhr).

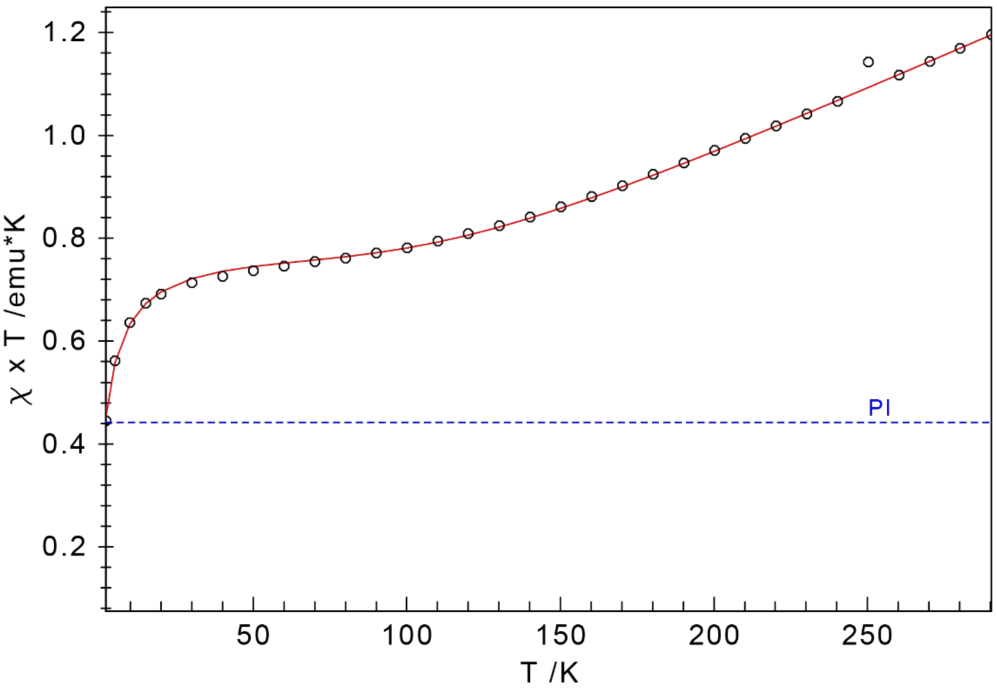

Figure S 15. Variable temperature dc magnetic susceptibility data of **2** collected under an applied magnetic field of 10000 Oe.

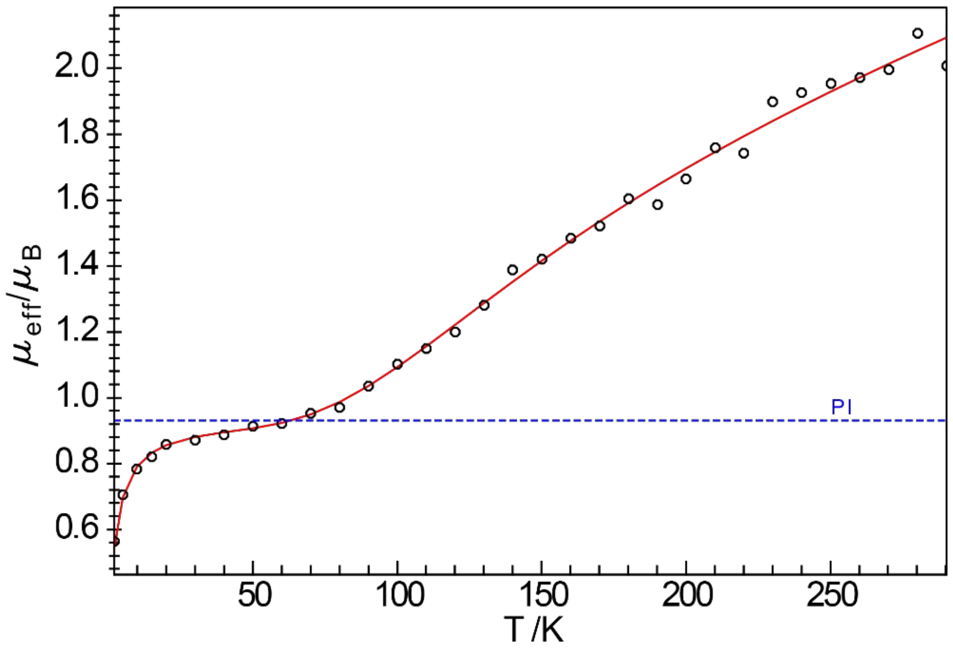

Figure S 16. Variable temperature dc magnetic susceptibility data of **3** collected under an applied magnetic field of 10000 Oe.

Table S 2. Magnetic SQUID data simulation parameters for compounds **2** and **3**

Compound	Spin of Nuclei	g-Values	TIP [emu]	Paramagnetic Impurity [%]	Coupling Const. J [cm^{-1}]
**2**	2.0 ; 2.5	2.0 ; 2.0	560 x 10-6	10.2 (S=2.5)	-118
**3**	2.0 ; 2.0	1.87 ; 1.87	300 x 10-6	2.7 (S=2.5)	-138

Crystallographic data

Low-temperature diffraction data (ω-scans) of complexes **1** and **3** were collected on a Bruker Nonius KappaCCD diffractometer with a Mo-target rotating-anode X-ray source and Incoatec Helios focusing multilayer optics and a Bruker Kappa Mach3 APEX-II diffractometer with a Bruker IµS and Incoatec Helios mirror (Mo Kα radiation; λ = 0.71073 Å). Data for **4** were collected on a Rigaku MicroMax-007HF diffractometer coupled to a Saturn994+ CCD detector with Cu Kα (λ = 1.54178 Å). All diffraction images were processed and scaled using Rigaku Oxford Diffraction software (CrysAlisPro; Rigaku OD: The Woodlands, TX, 2015). All non-hydrogen atoms were refined anisotropically. Hydrogen atoms were included in the model at geometrically calculated positions and refined using a riding model.

Compound **3** crystallized with 3.5 molecules of benzene, three on general positions and one on a center of inversion. Due to the disorder of the solvent molecules, their scattering contributions were removed using the program Platon/SQUEEZE.[6]

Table S 3 contains some crystallographic details of the refinements and CCDC numbers 2212975-2212977 contain full supplementary crystallographic data for **1**, **2** and **4**. These data can be obtained free of charge from The Cambridge Crystallographic Data Center via www.ccdc.cam.ac.uk/data_request/cif.

Table S 3. Crystallographic data for **1**, **3**, **4**.

Complex	**1**	**3**	**4**
CCDC number	2212975	2212977	2212976
Identification code	12128	11802SQ	007a-21137
Empirical formula	$C_{54}H_{50}P_4S_4Fe_2O$	$C_{57}H_{50}N_2P_4S_4Fe_2$	$C_{90}H_{112}N8Fe_2S_4P_4$
Formula weight	1078.76	1126.81	458.111
Temperature/K	100(2)	100(2)	100(2)
Crystal system	monoclinic	monoclinic	monoclinic
Space group	$P2_1/c$	$P2_1/c$	$P2_1/c$
a/Å	16.1761(12)	14.9674(13)	24.5586(7)
b/Å	17.577(2)	32.892(3)	17.5391(5)
c/Å	18.8685(13)	14.1941(12)	23.3734(8)
α/°	90	90	90
β/°	108.237(5)	101.611(2)	112.215(4)
γ/°	90	90	90
Volume/Å^3	5095.4(8)	6844.9(10)	9320.4(6)
Z	4	4	40
ρ_{calc}g/cm^3	1.406	1.093	3.265
μ/mm^{-1}	0.898	0.671	36.396
F(000)	2232	2328	9173.9
Crystal size/mm^3	0.18×0.1×0.08	0.10×0.08× 0.07	0.05 × 0.1 × 0.08
Radiation	MoKα (λ = 0.71073)	MoKα (λ = 0.71073)	Cu Kα (λ = 1.54184)
2Θ range for data collection/°	5.302 to 65	3.722 to 66.658	3.88 to 140.18
Reflections collected	102506	410921	287238
Independent reflections	18410 [R_{int} = 0.0600]	26353 [R_{int} = 0.0530]	16931 [R_{int} = 0.1031]
Data/restraints/parameters	18410/0/588	26353/108/625	16931/0/997
GooF^2	1.071	1.187	1.037
R_1 [I>2σ(I)]	0.0432	0.0779	0.0894
wR2 [I>σI)]	0.0802	0.1736	0.2399
R_1 (all data)	0.0708	0.0882	0.1146
wR_2 (all data)	0.0907	0.1799	0.2767
Largest diff. peak/hole/e Å^{-3}	0.90/-0.64	2.81/-1.53	1.63/-1.35

X-ray Absorption (XAS) and Emission (XES) Spectroscopy Methods

Resonant and non-resonant Kβ mainline XES and high energy resolution fluorescence detected (HERFD) XAS measurements were performed at beamline 6-2 of the Stanford synchrotron radiation lightsource (SSRL). Partial fluorescence yield (PFY) XAS measurements were performed at beamline 9-3 of the SSRL synchrotron. Measurements at both beamlines were performed according to the previously reported procedure.[7-9] XAS spectra were calibrated using an iron foil reference, with the first inflection point set to 7111.2 eV. XES spectra were calibrated by setting the maximum of the K$\beta_{1,3}$ line of an Fe_2O_3 reference to 7060.6 eV.

Raw PFY-XAS data were pre-processed via the ATHENA program, part of the Demeter software package.[10] Partial fluorescence yield (PFY) detected XAS scans were averaged together, and further pre- and post- edge background subtraction and the edge jump normalizations were applied. Raw HERFD-XAS and XES/RXES data were averaged using the program PyMCA.[11] HERFD-XAS data were further processed using the ATHENA program.[10] To compare PFY and HERFD-detected XAS, the HERFD spectra were normalized according to

$$I_{norm.\ HERFD} = \frac{I_{HERFD}}{\max\limits_{7000eV < x < 7400eV} I_{HERFD}} \max_{7000eV < x < 7400eV} I_{norm.\ XAS}.$$

Prior to normalization of the resonant and non-resonant XES spectra to the K$\beta_{1,3}$-maximum (set to one as previously published[12]), an offset correction was performed by averaging emission intensities starting from 7080 eV was applied using the following Python code (Code 1).

Code 1: Python function for converting XES into normalized XES spectra by using the Pandas library package for dataframes

```python
from typing import Tuple

import pandas as pd

def xes_norm(
    df: pd.DataFrame,
    eng: float = 7080,
    col_names: Tuple[str, str] = ("Energy", "Intensity"),
    norm_name: str = "Intensity_norm",
) -> pd.DataFrame:
    """Normalization of the resonant and non-resonant data.

    The normalization process is split into two parts:

    1. Offset correction for emission energies >7080 eV by dividing through the average
    2. Normalization by the maximum intensity of the resonant data.


    Args:
        df (pd.DataFrame): XES dataframe consists of energy and intensity
        eng (float): Start of the averaging industry; default to 7080.
        col_names (Tuple[str, str]): Name of the columns, first for energy and second
            for intensity; default to ("Energy", "Intensity").
        norm_name (str): Name of the column for normalized intensity; default to
            "Intensity_norm".

    Returns:
        pd.DataFrame: Adding a new column "Intensity_norm" to the dataframe.
    """
    df[norm_name] = df[col_names[1]] - df[df[col_names[0]] >= eng][col_names[1]].mean()
    df[norm_name] /= df[norm_name].max()
    return df
```

Numeric operations and plots are generated via the Python packages Pandas v1.4.3, NumPy v1.23.1, and matplotlib v3.5.2.[13-15] The first derivative of the **1**, **2**, and **3** are generated via *numpy.gradient*[1] module (Code 2) with non-homogeneous stepsize. The numerical gradient is calculated to the 1st order at the boundary condition, generally the approximation of $f_i^{(1)}$ is

$$\widehat{f_i^{(1)}} = \frac{h_s^2 f(x_i + h_d) + (h_d^2 - h_s^2) f(x_i) - h_d^2 f(x_i - h_s)}{h_s h_d (h_d + h_s)} + \mathcal{O}\left(\frac{h_d h_s^2 + h_s h_d^2}{h_d + h_s}\right).$$

The Code 1 and 2 is exported via CodeSnap[2].

[1] https://numpy.org/doc/stable/reference/generated/numpy.gradient.html
[2] https://github.com/kufii/CodeSnap

Code 2: Python function for generating the first derivative with non-homogeneous stepsize by using NumPy library package for array operations

```python
import numpy as np
import pandas as pd

def first_derivative(df: pd.DataFrame, col_x: str, col_y: str) -> np.ndarray:
    """Calculate the first derivative of a DataFrame column.

    Args:

        df (pd.DataFrame): DataFrame containing the data.
        col_x (str): Name of the column containing the x values.
        col_y (str): Name of the column containing the y values.

    Returns:

        np.ndarray: Array containing the first derivative of the y values.

    See Also:
        https://numpy.org/doc/stable/reference/generated/numpy.gradient.html
    """
    df = df.copy()
    return np.gradient(df[col_y], df[col_x], edge_order=1)
```

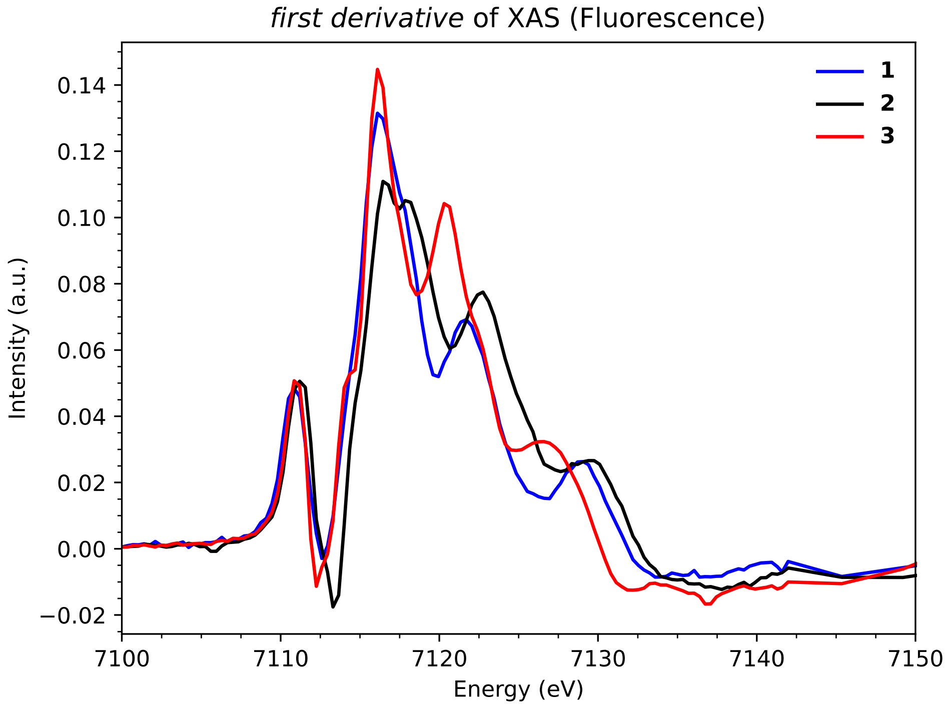

Figure S 17. First derivatives of the XAS spectra of complexes 1 (blue), 2 (black) and 3 (red).

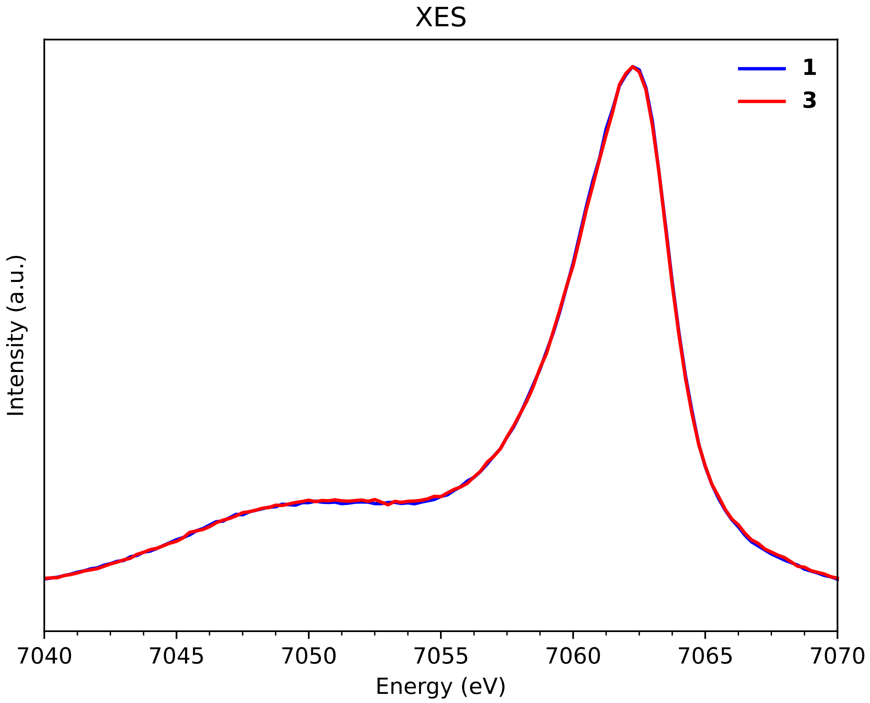

Figure S 18. Non-resonant Kβ XES of complex **1** (blue) and **3** (red).

References

1. Konu, J.; Chivers, T., Formation of a stable dicarbenoid and an unsaturated $C_2P_2S_2$ ring from two-electron oxidation of the $[C(PPh_2S)_2]^{2-}$ dianion. *Chem. Commun.,* **2008,** (40), 4995-4997.

2. Chávez, I.; Alvarez-Carena, A.; Molins*, E.; Roig, A.; Maniukiewicz, W.; Arancibia, A.; Arancibia, V.; Brand, H.; Manuel Manríquez*, J., Selective oxidants for organometallic compounds containing a stabilising anion of highly reactive cations: $(3,5(CF_3)_2C_6H_3)_4B^-)Cp_2Fe^+$ and $(3,5(CF_3)_2C_6H_3)_4B^-)Cp^*{}_2Fe^+$. *Journal of Organometallic Chemistry* **2000,** *601* (1), 126-132.

3. Kern, R. J., Tetrahydrofuran complexes of transition metal chlorides. *Journal of Inorganic and Nuclear Chemistry* **1962,** *24* (9), 1105-1109.

4. Fustier-Boutignon, M.; Heuclin, H.; Le Goff, X. F.; Mezailles, N., Transmetalation of a nucleophilic carbene fragment: from early to late transition metals. *Chem. Commun.* **2012,** *48* (27), 3306-8.

5. Bain, G. A.; Berry, J. F., Diamagnetic Corrections and Pascal's Constants. *Journal of Chemical Education* **2008,** *85* (4), 532.

6. Spek, A. L., PLATON SQUEEZE: a tool for the calculation of the disordered solvent contribution to the calculated structure factors. *Acta Crystallographica Section C: Structural Chemistry* **2015,** *71* (1), 9-18.

7. Van Stappen, C.; Jiménez-Vicente, E.; Pérez-González, A.; Yang, Z.-Y.; Seefeldt, L. C.; DeBeer, S.; Dean, D. R.; Decamps, L., A conformational role for NifW in the maturation of molybdenum nitrogenase P-cluster. *Chemical Science* **2022,** *13* (12), 3489-3500.

8. Kowalska, J. K.; Hahn, A. W.; Albers, A.; Schiewer, C. E.; Bjornsson, R.; Lima, F. A.; Meyer, F.; DeBeer, S., X-ray Absorption and Emission Spectroscopic Studies of $[L_2Fe_2S_2](n)$ Model Complexes: Implications for the Experimental Evaluation of Redox States in Iron-Sulfur Clusters. *Inorg. Chem.,* **2016,** ***55*** (9), 4485-97.

9. Lee, N.; Petrenko, T.; Bergmann, U.; Neese, F.; DeBeer, S., Probing Valence Orbital Composition with Iron Kβ X-ray Emission Spectroscopy. *J. Am. Chem. Soc.,* **2010,** *132* (28), 9715-9727.

10. Ravel, B.; Newville, M., ATHENA, ARTEMIS, HEPHAESTUS: data analysis for X-ray absorption spectroscopy using IFEFFIT. *J Synchrotron Radiat* **2005,** *12* (Pt 4), 537-41.

11. Solé, V. A.; Papillon, E.; Cotte, M.; Walter, P.; Susini, J., A multiplatform code for the analysis of energy-dispersive X-ray fluorescence spectra. *Spectrochimica Acta Part B: Atomic Spectroscopy* **2007,** *62* (1), 63-68.

12. Castillo, R. G.; Hahn, A. W.; Van Kuiken, B. E.; Henthorn, J. T.; McGale, J.; DeBeer, S., Probing Physical Oxidation State by Resonant X-ray Emission Spectroscopy: Applications to Iron Model Complexes and Nitrogenase. *Angew. Chem. Int. Ed.,***2021,** *60* (18), 10112-10121.

13. Reback, J.; McKinney, W.; Van Den Bossche, J.; Augspurger, T.; Cloud, P.; Klein, A.; Hawkins, S.; Roeschke, M.; Tratner, J.; She, C., pandas-dev/pandas: Pandas 1.0. 5. *Zenodo* **2020**.

14. Harris, C. R.; Millman, K. J.; van der Walt, S. J.; Gommers, R.; Virtanen, P.; Cournapeau, D.; Wieser, E.; Taylor, J.; Berg, S.; Smith, N. J.; Kern, R.; Picus, M.; Hoyer, S.; van Kerkwijk, M. H.; Brett, M.; Haldane, A.; Del Rio, J. F.; Wiebe, M.; Peterson, P.; Gerard-Marchant, P.; Sheppard, K.; Reddy, T.; Weckesser, W.; Abbasi, H.; Gohlke, C.; Oliphant, T. E., Array programming with NumPy. *Nature* **2020,** *585* (7825), 357-362.

15. Hunter, J. D., Matplotlib: A 2D Graphics Environment. *Computing in Science & Engineering* **2007,** *9* (3), 90-95.
